# Metagenomic analysis revealed a wide distribution of antibiotic resistance genes and biosynthesis of antibiotics in the gut of giant pandas

**DOI:** 10.1186/s12866-020-02078-x

**Published:** 2021-01-07

**Authors:** Ghulam Raza Mustafa, Caiwu Li, Siyue Zhao, Lei Jin, Xueping He, Muhammad Zubair Shabbir, Yongguo He, Ti Li, Wenwen Deng, Lin Xu, Yaowu Xiong, Guiquan Zhang, Hemin Zhang, Yan Huang, Likou Zou

**Affiliations:** 1 Department of Applied Microbiology, College of Resources, Sichuan Agricultural University, Chengdu, 611130 China; 2 Key Laboratory of State Forestry and Grassland Administration (SFGA) on Conservation Biology of Rare Animals in the Giant Panda National Park, The China Conservation and Research Center for the Giant Panda (CCRCGP), Dujiangyan, 611830 China; 3 Institute of Microbiology, The University of Veterinary and Animal Sciences, Lahore, 54600 Pakistan

**Keywords:** Gut microbiome, Antibiotic resistance gene, Biosynthesis of antibiotics, Giant panda, Metagenome

## Abstract

**Background:**

The gut microbiome is essential for the host’s health and serves as an essential reservoir of antibiotic resistance genes (ARGs). We investigated the effects of different factors, including the dietary shifts and age, on the functional characteristics of the giant panda’s gut microbiome (GPs) through shotgun metagenome sequencing. We explored the association between gut bacterial genera and ARGs within the gut based on network analysis.

**Results:**

Fecal samples (*n*=60) from captive juvenile, adult, and geriatric GPs were processed, and variations were identified in the gut microbiome according to different ages, the abundance of novel ARGs and the biosynthesis of antibiotics. Among 667 ARGs identified, nine from the top ten ARGs had a higher abundance in juveniles. For 102 ARGs against bacteria, a co-occurrence pattern revealed a positive association for predominant ARGs with *Streptococcus*. A comparative KEGG pathways analysis revealed an abundant biosynthesis of antibiotics among three different groups of GPs, where it was more significantly observed in the juvenile group. A co-occurrence pattern further revealed a positive association for the top ten ARGs, biosynthesis of antibiotics, and metabolic pathways.

**Conclusion:**

Gut of GPs serve as a reservoir for novel ARGs and biosynthesis of antibiotics. Dietary changes and age may influence the gut microbiome’s functional characteristics; however, it needs further studies to ascertain the study outcomes.

**Supplementary Information:**

The online version contains supplementary material available at 10.1186/s12866-020-02078-x.

## Background

One of the world’s most endangered animals, the Giant panda (GP) (*Ailuropoda melanoleuca*), is an endemic flagship species in China and a well-known symbol for wildlife conservation worldwide [1, 2]. It belongs to the family *Ursidae* (bears) and possesses the carnivore’s type of gastrointestinal tract, yet interestingly, subscribes to herbivores’ diet, consisting mainly of bamboo [3, 4]. The predominant causes of the decreasing range of GPs are their low nutritional intake, low fecundity, habitat destruction due to natural disasters, and human-associated activities [5–7]. Captive breeding centers are considered one of the main approaches to protect GPs and increase their population [8]. There are almost 21.8% of GPs are living in captivity [9].

Habitat affects the gut microbiomes of animals [10]. The microbiome resides in mammals’ gastrointestinal tracts are essential to maintain the host health [11–13]. The intestinal microbes depend on food, making them able to adopt the possible measures to modify the microbiome in the body and replace harmful bacteria to useful microbes [12]. As for as GPs are concerned, they spend up to 14 h of their daily time on feeding activities in captivity [13], consuming up to 12.5 to 14 kg of bamboo including stems, leaves, and shoots [14] as well as non-bamboo foods such as vegetable, fruits, high-fiber biscuits, [15], steamed bread [16], and commercial milk [17]. *Escherichia* and *Streptococcus* are the predominant members of the gut of GPs. Different species of *Streptococcus* are found in the gut of the GPs, i.e., *Streptococcus thermophilus* [18], which are known to produce a variety of negative factors such as antibiotic-like substances, bactericidal proteins, and metabolic end-products [19].

Diet is an essential factor that may influence the gut microbiome [20–24]. Increased antibiotic resistance genes (ARGs) have been observed with a high protein diet in a recent study [25]. Another study revealed a varying rate of expression of genes involved in phenylalanine metabolism, fatty acid biosynthesis, purine metabolism, glutathione metabolism, antibiotic resistance, and streptomycin biosynthesis in the gut of juvenile GPs that were fed with bamboo leaves and shoot as diet and the more genes were found in shooting stage [4]. Few ARGs such as beta-lactam, aminoglycoside, macrolides, and bacitracin were significantly higher in the gut microbiome of captive GPs than wild [9]. The rapidly emerging ARGs have been a global health concern with negative impacts [26] on animal and human health [27, 28]. Indeed, the gut microbiome is among the closely-related microbial communities on earth [29, 30] and serves as an essential reservoir of ARGs or gut resistome [31]. A recent study indicated genes corresponding to antibiotics’ biosynthesis and antibiotic resistance in the gut of juvenile GPs fed with the bamboo shoot [4]. Nevertheless, the study was unable to describe the complicated relationship between ARGs and the biosynthesis of antibiotics. With this background, applying a metagenome sequencing approach employed previously for other hosts’ originating microbiome [21, 32], we assessed 1) the role of a high abundance of *Streptococcus* in maintaining GPs intestinal microbiome balance, 2) proliferation of ARGs in the gut of GPs and their association with *Streptococcus*, and 3) KEGG pathways analysis to explore biosynthesis of antibiotics by the GPs’ gut microbiome.

## Results

### The overall abundance of antibiotic resistance genes (ARGs)

We extracted DNA from 60 fecal samples of captive GPs and analyzed gut microbiome composition and variations. Our results showed an abundance of various ARG types in the feces of GPs, where a total of 667 unique ARGs were identified. Among these, abundance of 570 ARGs ranged from 0.0001 to 3.6% had potential to confer resistance against a range of antibiotics such as Efflux pump (42%), Quinolones (13%), Peptide (12%), Aminocoumarines (7%), Fosfomycin (5%), Glycopeptide (4%), Lincosamide (4%), Beta-lactams (4%), Isoniazid (2%), Tetracyclines (2%), Aminoglycosides (1%), Diaminopyrimidine (1%), Macrolides (1%), Mupirocin (1%) etc. Each sample had varied ARGs. The relative abundance of ARGs in each of the fecal samples of GPs is presented (Additional file 1: Fig. S2a, Additional file 4: Fig. S2b).

**Fig. 1:**
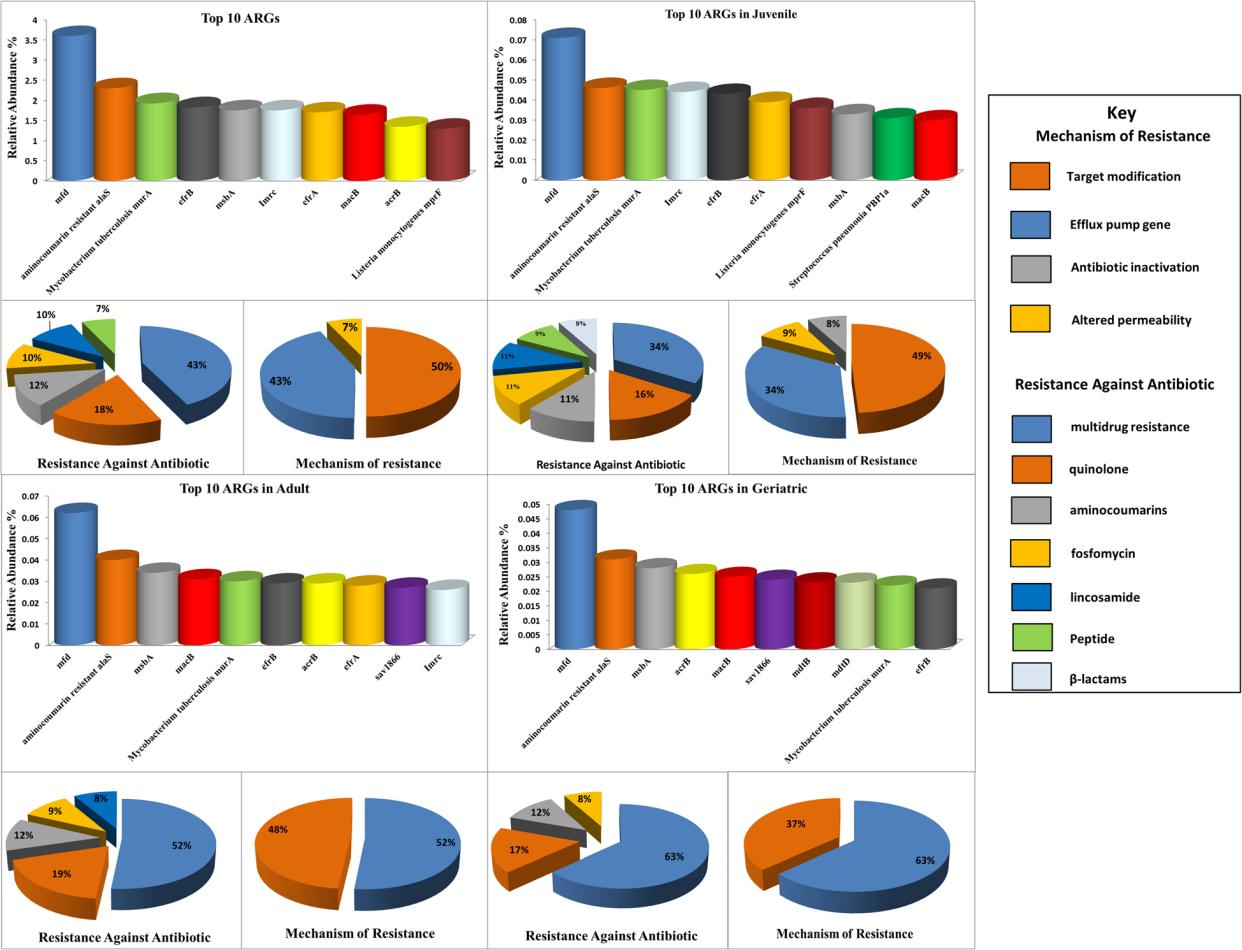
Classification of top-ten ARGs in all samples (**a**) according to their resistant antibiotics and resistance mechanism. Classification of top-ten ARGs as per families of resistant antibiotics and the mechanism of resistance in juveniles (**b**), adults (**c**), and geriatrics (**d**)

The *mfd* (3.6%), *aminocoumarin resistance alaS* (2.3%), *Mycobacterium tuberculosis murA* (1.93%)*, efrB* (1.83%)*, msbA* (1.8%)*, lmrc* (1.76%)*, efrA* (1.71%)*, macB* (1.65%)*, acrB* (1.35%)*,* and *Listeria monocytogenes mprF* (1.30%) were the most abundant ARGs in all samples. Genes (ARGs) corresponding to multi-drugs (43%), Quinolones (18%) via efflux pump (43%) and target modification (50%) were also identified. The *efrB* (1.83%)*, msbA* (1.8%)*, efrA* (1.71%)*, macB* (1.65%), and *acrB* (1.35%) were the most abundant multidrug resistance genes in all samples. The *mfd* that confers resistance to Quinolone was the most abundant ARG subtype. Aminocumarines (12%), Fosfomycin (10%), and Lincosomide (10%) revealed ARGs (*aminocoumarine resistant alaS, Mycobacterium tuberculosis murA*, and *lmrc*) that work through target modification. A peptide resistance gene, such as *Listeria monocytogenes mprF*, that works with altered permeability was also there in the ten most abundant ARGs. (Fig. 1a, Additional file 2: Table S1).

### Comparison of abundance of ARGs in different age groups

The ten most abundant gene types varied from group to group; however, the Quinolones resistance gene (*mfd*) that works via target modification was the most abundant in all groups. Some of the multidrug resistance genes were also common in all three groups. For example, geriatrics harbored a higher number of multidrug resistance genes, i.e., *efrb, macB, msbA, acrB, sav* 1866, *mdtB mdtD* those work via efflux pump. Interestingly, three of these ARGs (*efrB, macB,* and *msbA*) were common in all groups. The *sav* 1866 and *acrB* were common in adults and geriatrics; however, they were not among the top ten juveniles’ ARGs. In contrast, the gene *efrA*, which confers resistance to multi drugs via the efflux pump, was common in juveniles and adults but was not among the top ten ARGs of geriatrics. The Aminocoumarins and Fosfomycin resistance gene (*aminocoumarin-resistant alaS* and *Mycobacterium tuberculosis murA*) were the most abundant gene types of all the three groups. A lincosamide resistance gene (*Imrc*) was common and most abundant in the juveniles and adults but was not among the geriatrics’ top ten ARGs. Similarly, *Listeria monocytogenes mprF*, and *Streptococcus pneumonia PBP1a* were among the top ten ARGs of the juvenile group, but it was not the same for adults and geriatrics (Fig. 1b, c, d, Additional file 2: Table S1).

**Fig. 2:**
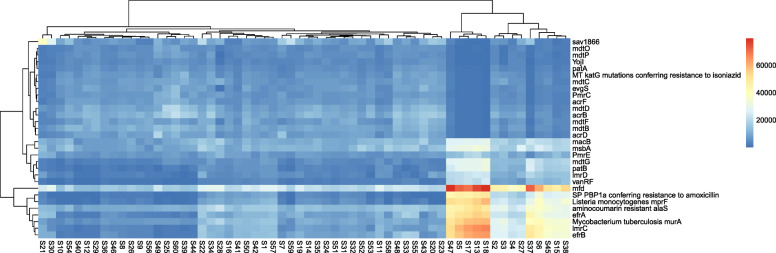
Heat-map shows the relative abundance of each gene-type in each of the individual of three different groups: The sample for juveniles, adults, and geriatrics are colored red, black, and blue, respectively. The gene-types (rows) and samples (columns) were clustered with R studio using the Spearman rank correlation and complete linkage. The indicator on the right denotes the relationship between the relative abundance and color range

Next, we mapped the top 30 ARGs to each individual in the different age groups of GPs. The *mfd* was the most abundant gene in the majority of the samples, particularly the S47 from the adult and S18 from the juvenile (4.5%), followed by S5, S6, S13, S17 (juveniles), S37, S38, and S45 (adults) (4.3 to 2.9%). However, a lower abundance of this particular gene (*mfd*) was observed in geriatrics. Some samples, such as S18, S13, S17, S5 (juveniles), and S47, (adult) had a high abundance of *lmrC* (7.1 to 5.6%)*, efrB* (6.7% to 5.4)*, Mycobacterium tuberculosis murA* (5.9 to 5.0%)*, Listeria monocytogenes mprF* (8.3 to 7.4%)*, efrA* (6.1 to 5.1%) and *aminocoumarin resistant alaS* (4.7 to 3.9%). However, it was contrary to S6, S15 (juveniles), S37, S45, and S38 (adults), where the abundance of these particular ARGs was relatively low and varied from 5.1 to 2.7%. Each sample had an abundance of ARGs that was different from each other. For example, S57 (geriatric) had 1.6% of *mfd,* S21 (adult) had 7.4% *sav* 1866, S60 (geriatric) had 3.6% *acrB* and 4.0% *mdtD* (Fig. 2, Additional file 1: Fig. S3).

**Fig. 3:**
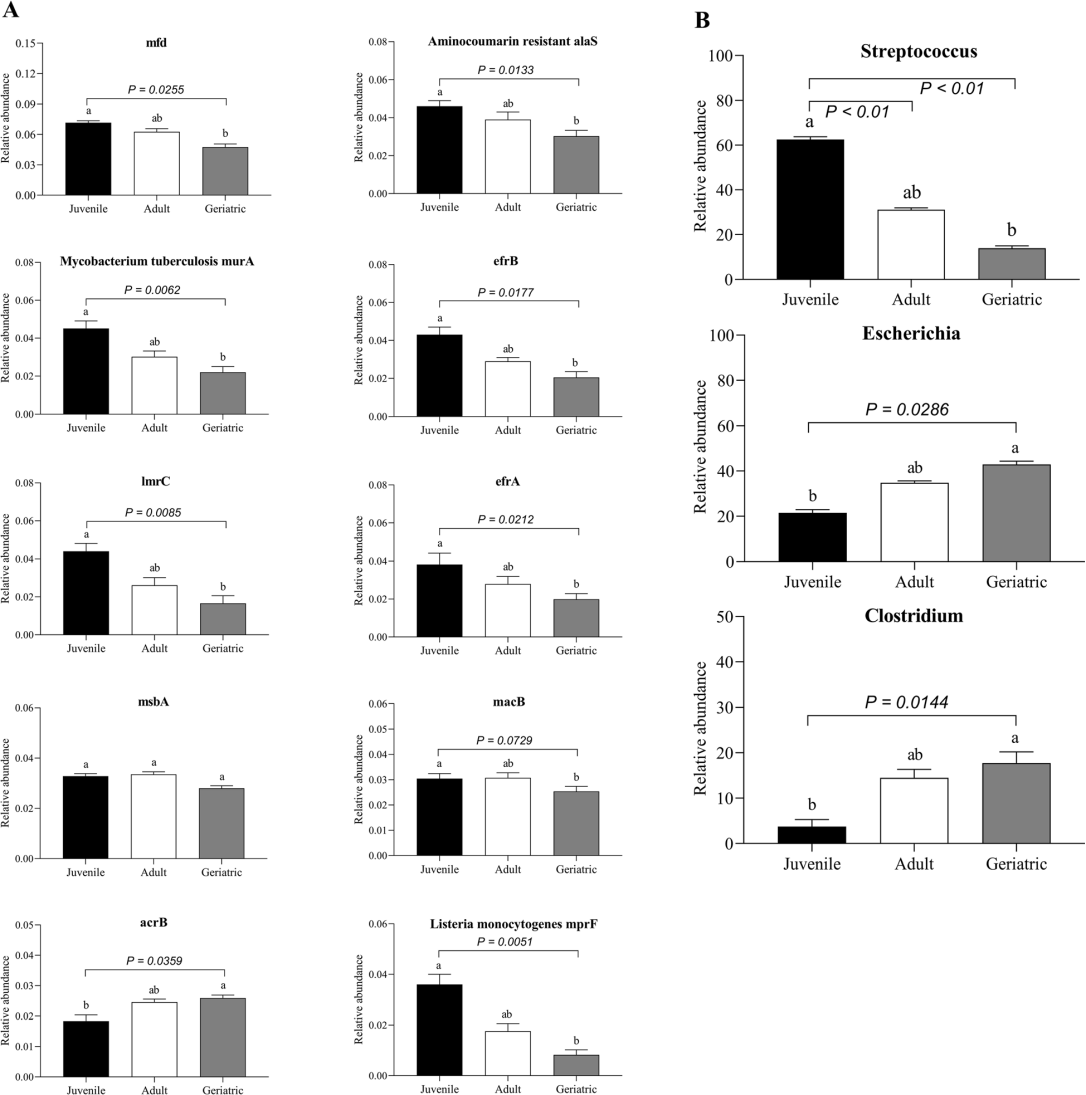
The relative abundance of top-ten ARGs (**a**) and top-three bacterial genera (**b**) in three different groups of GPs. The data are presented as the mean ± SE. Different lowercase letters indicate significant differences in relative abundance among three different groups of GPs. ^a-b^ Means with no common superscript are significantly different *P* < 0.05

ARGs exhibiting significant differences in relative abundance were identified (*P* < 0.05 and false discovery rate (FDR) = 0.062 selected threshold 0.05, while comparing the relative abundance of top ten ARGs among three groups of GPs, *mfd* gene was found most abundant (0.071%) in the juvenile group than adults (0.062%) and geriatrics (0.048%) (*P* = 0.0255). The *aminocoumarin resistance alas* (*P* = 0.0113)*, Mycobacterium tuberculosis murA* (*P* = 0.0062)*, efrB* (*P* = 0.0177)*, Imrc* (*P* = 0.0085)*, efrA* (*P* = 0.0212)*, acrB* (*P* = 0.0359) and *Listeria monocytogenes mprf* (*P* = 0.005) showed significant difference in abundance among the three groups. All the top ten ARGs showed significantly higher abundance in juveniles than adults and geriatrics. However, the gene *acrB*, was significantly more abundant in geriatrics than juveniles and adults. Though the genes *msbA* and *macB* had high abundance in juveniles; however, they were not statistically significant while comparing adults and geriatrics (Fig. 3a).

### The relative abundance of bacterial community in different age groups

An average abundance of approximately 20 genera in each sample was identified through metagenome analysis (Additional file 1: Fig. S4). Among these, *Streptococcus, Escherichia,* and *Clostridium* were the most frequent in all samples. *Streptococcus* occupied 31% of the total bacterial population in the gut microbiome, followed by *Escherichia* (24%) and *Clostridium* (8%). Concerning each group, the juvenile had a higher abundance of reads corresponding to *Streptococcus* (62%), followed by *Escherichia* (21%) and *Clostridium* (3%). *Escherichia* was the most abundant in adults (35%) and geriatrics (43%). *Streptococcus* (31%) and *Clostridium* (14%) were the other abundant genera in adults. On the other hand, for geriatrics, it was *Clostridium* (18%) and *Streptococcus* (14%) (Fig. 3b, Additional file 1: Fig. S5). A significant difference in the abundance of *Streptococcus*, *Escherichia,* and *Clostridium* was observed among three groups of GPs (*P* < 0.05 and false discovery rate (FDR) = 0.036 selected threshold 0.05. The abundance of *Streptococcus* in the juvenile was significantly more than in adult and geriatric (*P* = 0.008). Similarly, the abundance of *Escherichia* and *Clostridium* were significantly more geriatric than adults (*P* = 0.0286) and juveniles (*P* = 0.0144). The percentage of *Escherichia* was 21, 35, and 43%, while for *Clostridium,* it was found to be 3, 14, and 18%, respectively (Fig. 3b).

### Correlation analysis of bacterial genera and ARGs

**Fig. 4:**
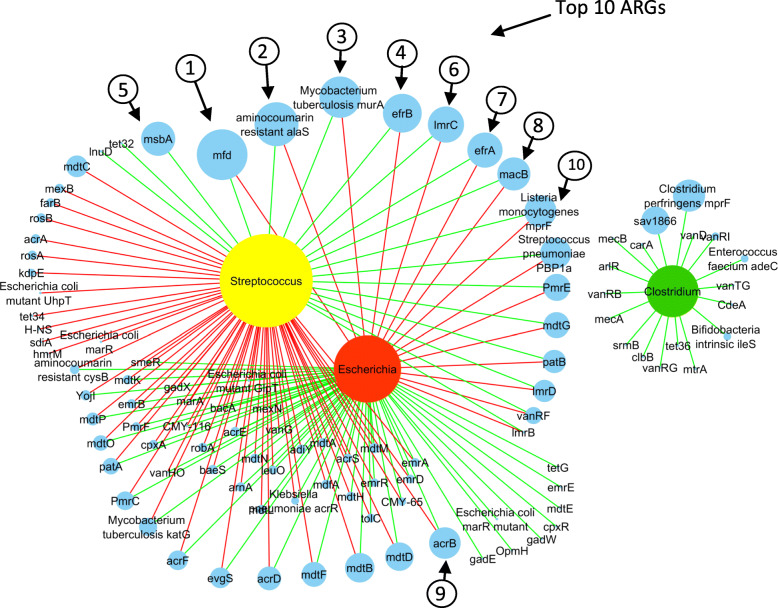
The network analysis is revealing the co-occurrence pattern between bacterial genera and ARGs. Yellow, red, green, and blue nodes represent *Streptococcus*, *Escherichia, Clostridium,* and ARGs. A connection represents a strong (Spearman’s correlation coefficient R^2^ > 0.8–0.9) and significant correlation (*P* < 0.05). Edges are colored according to the correlation coefficient, where green represents positive, and red represent the negative correlation. Node size corresponds to the relative abundance of ARGs and bacterial genera

Based on the Pearson coefficient of correlation, with an average abundance of ≥ 0.01%, we analyzed the correlation among 84 genera. We observed positive correlations between ARGs and the majority of bacterial genera (Additional file 1: Fig. S6). We constructed a network of correlations between the abundance of bacterial genera and ARGs from all samples to predict co-occurrence among top-three bacterial genera and ARGs. In the network, we compared three bacteria at the genus level against 102 ARGs, where each node is presented either by a bacterial genus or ARG in the resulting network of significant interactions. The network included 105 nodes and 164 edges. The correlations identified by the network were predominantly positive. In *Streptococcus* and *Escherichia* communities, we found a higher number of negatively correlated ARGs (*n*=59) with *Streptococcus,* whereas *Escherichia* had a positive correlation with most of the ARGs (*n*=54). A total of 18 ARGs were found positively correlated with *Streptococcus,* and interestingly, most of these were from the most abundant ARGs. However, for *Escherichia*, they were negatively correlated (Fig. 4). The *mfd* was positively correlated with *Streptococcus* (r = 0.97), while it was correlated otherwise with *Escherichia* (r = − 0.71). *aminocoumarin resistance alaS* (r = 0.97), *Mycobacterium tuberculosis murA* (r = 0.99), *efrB* (r = 0.98), *lmrC* (r = 0.99), *efrA* (r = 0.96), *macB* (r = 0.90), *Listeria monocytogenes mprF* (r = 0.99), and *Streptococcus pneumonia PBP1a* (r = 0.99) were positively correlated with *Streptococcus*. On the other hand, *acrB* (r = 0.69), *mdtD* (r = 0.61), *mdtB* (r = 0.84), *mdtF* (r = 0.83) and *acrD* (r = 0.88) showed positive correlation with *Escherichia. Clostridium* showed a positive correlation with all 18 ARGs (Fig. 4, Additional file 2: Table S3).

### The abundance of pathways in biosynthesis of antibiotics, metabolism, and antimicrobial resistance

**Fig. 5:**
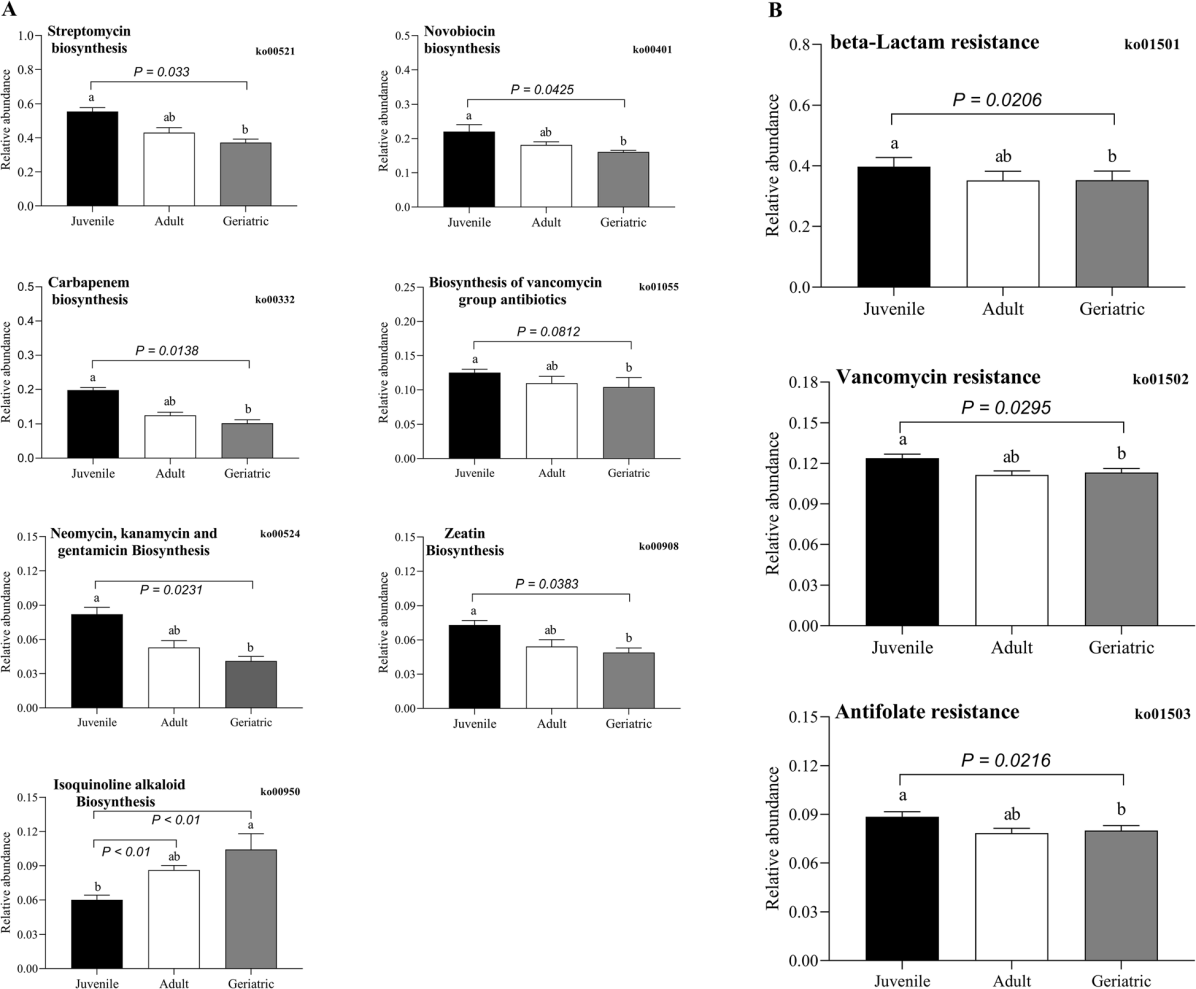
The relative abundance of KEGG pathways-based biosynthesis of antibiotics (**a**) and resistance (**b**) in three different groups of GPs. The data are presented as the mean ± SE. Different lowercase letters indicate significant differences in relative abundance among three different groups of GPs. ^a-b^ Means with no common superscript are significantly different *P* < 0.05

**Fig. 6:**
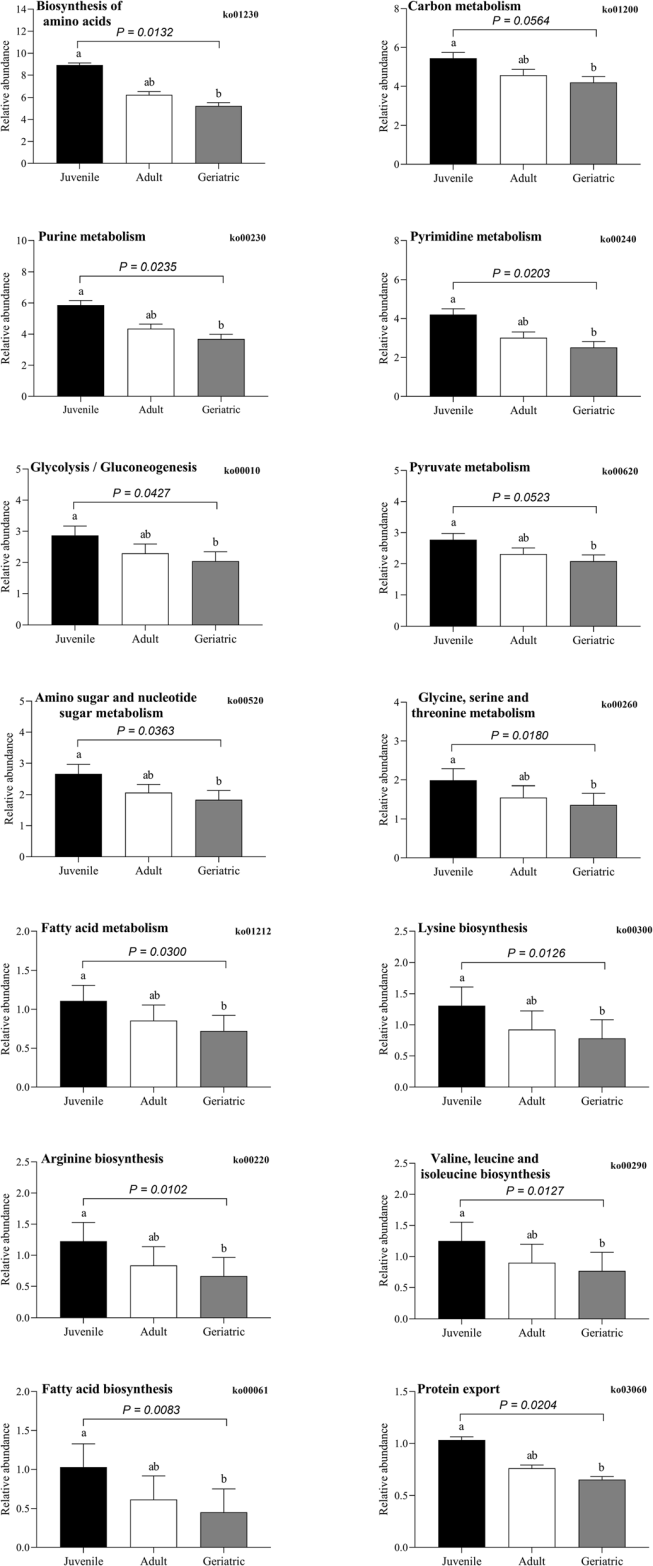
The relative abundance of KEGG metabolic pathways-based biosynthesis of biologic substances other than antibiotics in three different groups of GPs. The data are presented as the mean ± SE. Different lowercase letters indicate significant differences of relative abundance among three different groups of GPs. ^a-b^ Means with no common superscript are significantly different *P* < 0.05

We determined the functional annotation for the biosynthesis of antibiotics, metabolic and antimicrobial pathways using the KEGG database. Different groups of GPs revealed significant differences in their abundance while comparing KEGG pathways attribute. The key difference supported by the statistical significance was determined in terms of abundance of metabolic functions related to antibiotic biosynthesis. For instance, KEGG pathways-based metabolic function varied for biosynthesis of different antibiotics such as streptomycin (ko=00521, *P* = 0.033), novobiocin (ko=00401, *P* = 0.0425), carbapenem (ko=00332, *P* = 0.0138), vancomycin (ko=01055, *P =* 0.0812), neomycin, kanamycin and gentamicin (ko=00524, *P* = 0.0231), and zeatin (ko=00908, *P* = 0.0383) were more abundant in juveniles than adults and geriatrics. Conversely, biosynthesis related to Isoquinoline alkaloid (ko=00950, *P* = 0.0068) showed a significantly higher abundance in geriatrics and adults than juveniles (Fig. 5a, Additional file 1: Fig. S9A). Likewise, metabolic function related to biosynthesis of amino acid (ko=01230, *P =* 0.0123), carbon (ko=01200, *P =* 0.0564), purine (ko=00230, *P =* 0.0235), pyrimidine (ko=00240, *P =* 0.0203), glycolysis / gluconeogenesis (ko=00010, *P =* 0.0427), pyruvate (ko=00620, *P =* 0.0300), amino and nucleotide sugar (ko=00520, *P =* 0.0363), glycine, serine and threonine each (ko=00260, *P =* 0.0180), fatty acid (ko=01212, *P =* 0.0123), lysine (ko=00300, *P =* 0.0126), arginine (ko=00220, *P =* 0.0102), valine, leucine and isoleucine each (ko=00290, *P =* 0.0127), protein export (ko=03060, *P =* 0.0204), and fatty acid (ko=00061, *P =* 0.0083) were more abundant in juveniles than others (Fig. 6, Additional file 1: Fig. S7). A few differences in antimicrobial resistance were also revealed by KEGG pathway analysis. For instance, there was a significant higher abundance of resistance for beta-lactam antibiotics (ko=01501, *P* = 0.0206), vancomycin (ko=01502, *P* = 0.0295), and antifolate (ko=01523, *P* = 0.0216) in juveniles than adults and geriatrics (Fig. 5b, Additional file 1: Fig. S9B).

### Correlation analysis of pathways in biosynthesis of antibiotic, metabolism, and ARGs

**Fig. 7:**
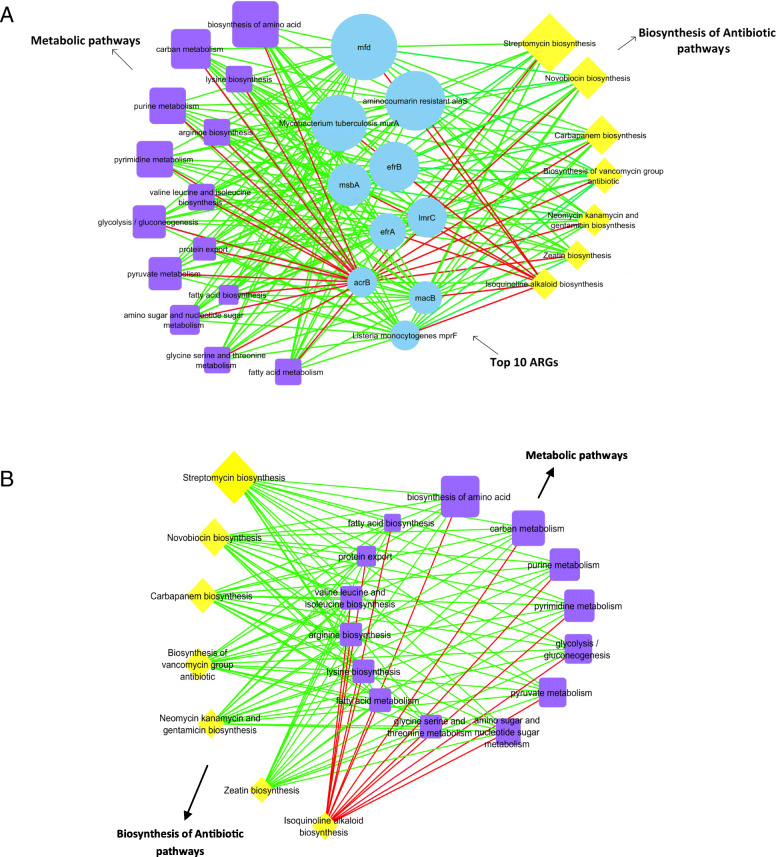
The network analysis reveals the co-occurrence patterns between metabolic pathways, pathways in biosynthesis of antibiotics and ARGs (**a**), metabolic pathways, and pathways in biosynthesis of antibiotics (**b**). Yellow, blue, and purple nodes represent biosynthesis of antibiotics, ARGs*,* and metabolic pathways*,* respectively. A connection represents a strong (Spearman’s correlation coefficient R^2^ > 0.8–0.9) and significant (*P* < 0.05) correlation. Edges are colored according to the correlation coefficient (green and red color represent positive and negative correlation), and node size corresponds to the relative abundance of metabolic pathways, pathways in biosynthesis of antibiotics and ARGs

Based upon the Pearson coefficient of correlation, we analyzed the correlation between the abundance of top-ten ARGs, pathways involved in antibiotics’ biosynthesis, and their metabolism. We compared the top ten ARGs against 14 metabolic pathways and 7 pathways in antibiotics’ biosynthesis in the network. We observed positive correlations between the majority of ARGs and metabolic pathways as well as ARGs and pathways in the biosynthesis of antibiotics. The network included 31 nodes and 210 edges. Except for *acrB,* all the top-ten ARGs were positively correlated with metabolic pathways and pathways in antibiotics biosynthesis*.* The said ARG was negatively correlated with all the metabolic pathways, and six of the antibiotics’ biosynthesis pathways had an exclusive positive correlation with isoquinoline alkaloid biosynthesis (Fig. 7a). We constructed a network of correlations between the abundance of these pathways from all samples to predict co-occurrence among the biosynthesis pathways of antibiotics and metabolism. The network compared 14 metabolic pathways against 7 pathways in the biosynthesis of antibiotics. The correlations identified by the network were predominantly positive. All the pathways in the biosynthesis of antibiotics were positively correlated with metabolic pathways, except for isoquinoline alkaloid biosynthesis (Fig. 7a and b, Additional file 2: Table S4).

### Antimicrobial susceptibility in *Streptococcus*

The antimicrobial susceptibility patterns of all the isolates of *Streptococcus* are presented (Additional file 3: Table S2a, b). All the isolates showed a lack of resistance to any of the studied antimicrobials. All 11 strains showed 100% susceptibility to beta-lactam, lincosamides, glycopeptides, miscellaneous, and carbapenems. Some strains with intermediate susceptibility to some antimicrobial were also found. For instance, an intermediate susceptibility was observed for strain 1 to azithromycin, ofloxacin, and erythromycin. Strain 2 had intermediate susceptibility to azithromycin, levofloxacin, and ofloxacin, strain 3 and 4 to levofloxacin and ofloxacin), strain 6 and 7 to ofloxacin, strain 8 and 9 to azithromycin and ofloxacin, while strain 10 had intermediate susceptibility to rifampicin and levofloxacin.

## Discussion

The GP, the iconic species and national treasure of China, is well-known for its unique dietary habits, attractive appearance, and decreasing population. Substantial evidence highlights the importance of the gut microbiome in animals’ health [33–35]. Several studies have reported on the gut microbes of GP or pathways (degrading pathways, metabolic pathways etc.) in recent years [4, 18, 36]. Each study’s primary focus was GP cubs’ gut microbiome and a specific link between the gut microbiome and pathways (Xue et al. 2015, Zhang et al., 2018). Tun et al. [15] compared the microbial compositions of GPs of different ages (adults and geriatrics) and found that the older ones possess a lower bacterial species richness and diversity and carry novel homoacetogens in their gut. Xue et al. [16] focused on diversity and the gut microbiome structure, particularly during the extreme seasonal variations in the gut of cubs, juvenile, and adult GPs. They reported excessive variations in the gut microbiome of GPs across different seasons and ages and observed an impact of seasonal availability of diet (bamboo parts) on the gut microbiome. Zhang et al. [4] studied the influence of diet and dietary changes on the gut microbiome in different age groups gut microbiome. They reported that GPs’ gut microbiome is not well-adapted to the degradation of cellulose and lignin in their highly fibrous diet (bamboo); however, they have acclimatized to more readily digestible carbohydrates to have the most out of nutritional and energetic intake from bamboo. Nevertheless, there is a paucity of research on the gut microbiome’s potential for the biosynthesis of antibiotics, the ARGs, and the importance of *Streptococcus* in the gut and its association with ARGs. We performed metagenomic sequencing analysis of the gut of the GPs, allowing deep insight into the gut microbiome, variation within the microbiome, and their potential to the biosynthesis of antibiotics, the ARGs, and their association with the resident microbiome.

Previous findings suggested that *Enterobacteracea* and *Streptococcae* dominate the GP’s microbiome during the first 2 months of age; however, it is dominated by 3rd to 12th-month *Lactobacillae* and *Clostridiaceae* [4]. We identified variations within the GP’s gut across different ages with a reduced abundance of *Streptococcus* and an increased abundance of *Escherichia* by age (Additional file 1: Fig. S8). Our findings showed a significantly higher abundance of *Streptococcus* in the juvenile than adult and geriatric. In contrast, *Escherichia,* another predominant member of GP’s gut microbiome, [16, 18, 37] showed significantly higher abundance in geriatrics than adults and juveniles. This variation may indicate the negative and positive association of bacteria with age where *Streptococcus* has a positive association from birth to juvenile, while *Escherichia* has otherwise [4]. *Streptococcus* showed a negative relationship with ages from juvenile to adult and from adult to geriatric. On the other hand, *Escherichia* has a positive relationship from juvenile to adult and from adult to geriatric (Additional file 1: Fig. S8). *Escherichia* can also be speculated to decrease *Streptococcus’s* decreasing abundance in adult and geriatric GPs, but there lacks a shred of strong evidence for this hypothesis. Therefore, further investigation should be done to observe its decreasing abundance in adult and geriatric. The use of *Streptococcus* in feed supplement of adult and geriatric GPs as probiotic may meet that expectation to find out whether *Escherichia* also suppresses *Streptococcus* or the age factor is the only cause for the reducing abundance of *Streptococcus*. This is important because a previous study demonstrated that the addition of a *Streptococcus*-carrying-probiotic reduced enterotoxigenic *E.coli* colonization in swine [38, 39].

*Streptococcus,* particularly the *Streptococcus thermophilus,* is the most abundant species in the gut of GPs [18]. We also observed a dominance of *Streptococcus* among juveniles, which suggests a beneficial impact on animal health. It possesses inhibitory effects on the growth of gut-associated pathogens and, therefore, is known to keep the host healthy. In this regard, diet can be a factor that influences animal gut microbiome [22, 36] and may potentially contribute to the abundance of *Streptococcus* in the juvenile group. This is simply because juveniles’ diet in captivity is fermented milk, steamed bread, bamboo shoot, and leaves [4, 40]. *Streptococcus thermophilus* comes from fermented dairy products, e.g., fermented milk [19]. Here we demonstrate that *Streptococcus’* addition as a probiotic in geriatrics diet that showed a reduced abundance of *Streptococcus* (Additional file 1: Fig. S8) might improve the GPs’ health by maintaining its microbiome’s balance within the intestine. It has been reported that the use of probiotic drink containing *Streptococcus thermophilus* along with other bacteria with similar properties may prevent diarrhea and subsequently decreased morbidity and mortality if used regularly in the patients of age greater than 50 [41]. However, further investigation should be done to study these genera’s roles in the gut of GPs and the potential impact on the abundance of *Streptococcus* and *Escherichia* in the guts of juvenile, adult, and geriatric. This is perhaps the first attempt to explore the abundance of ARGs and their association with bacteria in the gut of GPs through metagenome sequencing.

The gut microbiome is among the most densely colonized microbial communities on earth [29], and serve as anessential reservoir of ARGs, referred to as the gut resistome [31]. A few studies [4, 9] have reported ARGs in the gut of captive GPs, but these studies have not provided many details about these ARGs. The present study showed an abundance of ARGs in the gut of three groups of GPs, where the top-most ARGs included *mfd, aminocoumarin resistance alas, Mycobacterium tuberculosis murA, efrB, lmrc, efrA, msbA, macB, acrB,* and *Listeria monocytogenes mprf.* The abundance of these ARGs in the gut could be speculated through various routes. Contaminated water can be an essential source for the spread of ARGs [42]. Found in the gut of the captive GPs, the aminoglycoside, bacitracin, and beta-lactam resistance genes are the most abundant in the drinking water [43]. The ARGs related to beta-lactam are also found in the gut of juvenile GPs in our study, suggesting that contaminated water may be one source for these genes’ presence. The GPs living in captivity monitored frequently and received regular medical care, which can provide selective pressure for the acquisition of ARGs in the gut of captive GPs and therefore taken as a possible factor for the contribution in an increased abundance of ARGs [9]. Another vital route for the spread of these ARGs is air pollution that has risen remarkably in recent years in the Sichuan province [44, 45]. Human contact with GPs in captivity could be taken as another source for these ARGs [43, 46]. Inheritance can also be a possible factor for the spread of ARGs, and in the subject matter, vertical transmission of the gut microbiome has been evidenced [47]. Owing to these speculations and evidence there lies a severe ‘GPs’ health-related concern for these ARGs, and therefore necessary interventions and future research should be continued on these aspects.

Concerning the abundance of ARGs, we identified excessive variations within the gut microbiome of GPs across different age groups, which may exemplify the association between bacteria and ARGs. The network analysis revealed a strong association of ARGs with bacteria. We found that ten top-most abundant ARGs positively associate with *Streptococcus*, except for *acrB,* which is positively associated with *Escherichia* (Fig. 4). It is noteworthy that overall results from the metagenomics-based analysis of ARGs and *Streptococcus* showed similar trends where the abundance of these ARGs decreased with the decreasing abundance of *Streptococcus* by age in adults and geriatrics, except for *acrB,* which increased by age along with *Escherichia* (Fig. 3a and b). Such a pattern suggests that some ARGs may play a role in bacteria’s survival within the juvenile’s gut. Previously, it has been evidenced that the predominant ARG (the *mfd*) in the gut of juvenile plays a vital role in the survival of Gram-positive as well as Gram-negative bacteria in humans [48]. Based on *Streptococcus* and *mfd’s* association, we observed that *mfd* contribute to *Streptococcus’s* survival in the juvenile group in our study. *Streptococcus’s* strong association and ARGs can be due to *Streptococcus’s* ability to produce hydrogen peroxide (H_2_O_2_) that inhibits other microorganisms’ growth in the gut [49–51]. An increased abundance of *mfd* can also be due to *Streptococcus’s* particular property because *mfd* has been found resistant to H_2_O_2_ [52]. Hence, here we hypothesized that other bacteria in the gut might also be producing *mfd* for their survival, and perhaps this is the reason for an increased abundance of *mfd* in all samples of the study. Further, we tested the antimicrobial susceptibility of 11 strains of *Streptococcus* against 13 antibiotics by the disk diffusion method, where most of the strains (*n*=7) were from the juvenile group. Intriguingly, all the strains were found susceptible to antimicrobial drugs (Additional file 3, Table S2a, and b). The antimicrobial susceptibility testing confirmed that *Streptococcus* is sensitive to ARGs and that there is an association between ARGs and *Streptococcus* that can help the survival of the gut microbiome.

Concerning the abundance of ARGs in different age groups, we found an increasing trend by age in the reads corresponding to multidrug resistance genes via the efflux pump. Intestinal infirmities have mostly been recognized in geriatrics GPs [53], generally ascribed to the more significant number of multidrug resistance genes. The primary reason could be an abundance of *Escherichia* since a previous study proved that the genes resistant to multi drugs decreased with *Escherichia’s* inhibition [54]. We also found a multidrug resistance gene (*acrB)* that had a positive association with *Escherichia.* Interestingly, *acrB,* an efflux membrane transporter that conferred resistance to several antibiotics classes, negatively associated with diet and was not among the top-ten ARGs of juveniles. Efflux pumps are a part of bacteria’s intrinsic resistance mechanism and decrease different antibiotic concentrations within the cell wall [55]. Since efflux pumps can be transferred horizontally [56], the presence of *acrB* in adults and geriatrics could be involved in horizontal gene transfer events [57].

An abundance of 280 KEGG orthologous was found in the gut of GP’s known to be involved in the biosynthesis of antibiotics and several metabolic pathways. (Fig. 6, Additional file 1: Fig. S7). The biosynthesis of antibiotics and metabolic pathways were significantly higher in the juvenile group, which may correspond to a higher relative abundance of ARGs and positive associations between ARGs and *Streptococcus*. The diet of GP could be considered an essential source for the biosynthesis of antibiotics simply because we found a positive association between metabolic pathways and pathways in biosynthesis of antibiotics with top-ten ARGs (Fig. 7a), and metabolic pathways with pathways in biosynthesis of antibiotics (Fig. 7b).

**Fig. 8:**
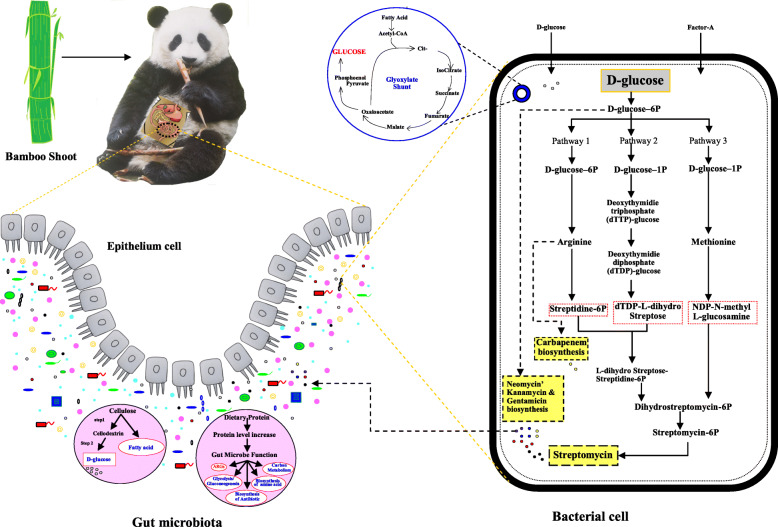
Effects of dietary changes of GP, such as increased level of protein, alter the gut microbiome function i.e. increased abundance of ARGs, glycolysis / gluconeogenesis, metabolism for carbon, fatty acid, and biosynthesis of antibiotic, amino acid in juveniles. The picture of panda (Qiaoyi), from the China Conservation and Research Center for the Giant Panda has been used in this figure

Increased biosynthesis of antibiotics in the juvenile group suggests dietary shifts from milk to bamboo leaves and shoots. Previous studies showed that the number of genes related to antibiotics’ biosynthesis increased from birth (milk) to the juvenile stage when fed with bamboo shoots than leaves [4]. Only one bamboo species is provided to GP’s in captivity, where they primarily eat shoot in spring and leaves in summer. Contrary to this, wild GP has a provision of more than one kind of bamboo species for most time of a year [58]. The nutritional values in different parts of bamboo vary significantly [59–62]. Consumption of single-parts for an extended period may lead to nutritional imbalance and may have a negative effect on GP’s health [63]. For instance, in a previous study, a higher protein concentration was found in bamboo’s shooting stage than the leaf stage [37]. Long-term consumption of bamboo shoots with high protein may affect the function of GP’s gut microbiome and, subsequently, the biosynthesis of antibiotics. The genes abundant in juvenile included those that are involved in fatty acid biosynthesis, phenylalanine metabolism, purine metabolism, glutathione metabolism, antibiotic resistance, and biosynthesis of streptomycin in the shooting stage [4]. The dietary nutritional content, especially protein content, was previously found associated with the abundance of ARGs in the gut [25]. Besides milk, bamboo shoots are a good protein source with protein content ranging from 1.49 g/100 g to 4.04 g/100 g in fresh bamboo shoots [64, 65]. Although protein intake is essential for increasing body mass, a high protein level in the gut may increase the bioavailability of glucose, amino acid, and fatty acid [63]. The higher protein level, particularly undigested protein, supports pathogens and protein-fermenting bacteria to increase health problems. This type of change of the gut microbiome can affect the gut hindrance and immune system by regulating gene expression in relevant signaling pathways and those involved in secretion of metabolites [66]. We delineate that the dietary changes of GP, such as increased level of protein, alter the gut microbiome function i.e. increased abundance of glycolysis / gluconeogenesis, protein export, metabolism for carbon, fatty acid, glycine, lysine, and biosynthesis of antibiotic, amino acid and fatty acid in juveniles (Fig. 8). Cumulative evidence showed that intake of excessive protein could adversely affect the health, therefore a diet containing a suitable ratio between protein and carbohydrate is recommended [66, 67]. A balanced diet should be provided to GPs offering more than one part of bamboo throughout the year or decreasing the level of protein in bamboo shoots [68, 69].

As per annotation by the KEGG database, streptomycin’s biosynthesis was the most abundant in all groups (Fig. 5a). It was significantly higher in juveniles than in the other two groups. Though it needs to be further investigated, antibiotics’ biosynthesis is speculated to be due to *Streptomyces*. The antibiotics produced by *Streptomyces* are typically secondary metabolites produced at the end of the exponential growth [70]. The biosynthesis of streptomycin is a complex process. For instance, the three parts of the streptomycin molecule, (a) streptidine (b) streptose, and (c) N-methyl glucosamine are formed by three different pathways and then are joined together to form the antibiotic (Fig. 8). Production of this antibiotic in *S. griseus* is regulated by an inducer called Factor-A, which triggers streptomycin biosynthesis at a low concentration [71]. A study reported that enzymes, uniquely over-expressed in the shooting stage, were involved in cellulose and hemicellulose degradation (Fig. 8) to cellodextrin followed by conversion to D-glucose (release of energy) [37]. The D-glucose is an excellent precursor of all the carbon atoms of streptomycin and is utilized by the *S. griseus* for streptomycin biosynthesis along with glycine [72]. Resistant pathways annotated by KEGG in our study suggest that ARGs in the gut of GPs are due to the biosynthesis of antibiotics.

Captivity (a human-constructed environment) represents an extreme change from the living environment in the wild. In captivity, the animals experience many changes, including reduced contact with different types of habitat, veterinary medical interventions, abruptly reduced range, reduced interactions with other species, changes or limitations in diet, and increased contact with human-associated microbes. Together these interventions may affect the gut microbiome [73] behavior [74, 75], and the health of the animal [75, 76]. In captivity, GPs live in a well-defined home range individually according to its solitary nature. Our study found the differences in ARGs’ abundance within our investigated GP’s population with the same social structure through heatmap analysis of the top 30 ARGs to each individual of different age groups. Some samples had more abundance of ARGs, while others had a low abundance. We observed a significant high abundance of ARGs in the samples from the juvenile group, which may be linked to the less adaptation to habitat. However, individual differences also exist since we found some samples from adults with much abundance of ARGs. An animal’s health and its ability to adapt to a situation, environment, or habitat depend upon a combination of its genetic makeup, which defines physical and psychological characteristics and its experience with its environment [77].

Age is generally considered an essential factor in shaping the gut microbiome. The rapid growth of juveniles and their dietary shift negatively affect the gut microbiome composition, and the more extensive interaction with the diet is essential for the maturity of the gut microbiome [78] of juvenile GPs. The maturity of the gut microbiome generally accelerates at the juvenile stage when environmental factors (e.g., diet) intervene with solid food [78] (bamboo shoot). A shift in the gut of juveniles increased the genes related to the biosynthesis of antibiotics and ARGs. The decreasing level of biosynthesis of antibiotics in adults and geriatrics can be due to the adaptation of its gut microbiome structure to the bamboo diet. This hypothesis/structure should further be ascertained to evaluate other factors that may decrease antibiotic biosynthesis in adults and geriatrics GPs.

The study had the limitation. One of the significant limitations was the inherent ability of metagenomics pipelines used in this study, where a maximum resolution for reads corresponding to a particular taxonomic node was limited to the level of genus. It would have been ideal if the relationship among ARGs could be made at the level of species; however, ARGs’ relationship was limited to genera nodes. This is becausenot all the environmental microorganisms are yet identified at the species level in the public database. Also, there are inherited limitations of the metagenomic pipelines (e.g., used in this study) where the partial gene sequence recovered through shotgun sequencing may not accurately delineate the recovered sequences to the species node in the publicly available database. Thus, the study outcomes should be taken and considered accordingly. Nevertheless, future studies that can elucidate the relationship between ARGs and the species are needed to ascertain further the outcomes presented herein for GPs under captivity.

## Conclusion

This study provides insight into GP’s gut microbiome’s potential to possess novel ARGs and the potential for antibiotics’ biosynthesis. The study reveals that dietary changes and the age of GPs may influence the resident gut microbiome’s functional characteristics in terms of the biosynthesis of antibiotics and an increased/reduced abundance of ARGs. Further studies are required to elucidate the relationship between dietary changes and ARGs as well as the potential linkage between the gut microbiome function and animal’s health.

## Methods

### Source of Giant panda

The animals were housed in the China Conservation and Research Center for the Giant Panda (CCRCGP), Sichuan, China. The experimental procedures complied with the current laws on animal welfare and research in China. The CCRCGP approved all sample collection protocols in this study.

### Sample collection

We collected 60 fecal samples from healthy captive GPs of different age groups devoid of antibiotics administration. Samples included 19 juveniles (aged 2 to 4 years), 35 adults (aged 5 to 19 years), and 6 geriatrics (aged 21 to 24 years) (Additional file 1: Fig. S1). Approximately15–30 kg bamboo, including stems and leaves, were fed to giant pandas every day. Besides, 3–8 kg shoots, 0.5–1.6 kg steamed bread, 300 g apples, and 300 g carrots were provided to each juvenile GP. In this group, the bamboo was dominated by bamboo leaves. Two-year-old giant pandas also received 500 mL of milk per feeding. For adults, 5–10 kg shoots, 1–1.8 kg steamed bread, 500 g apples, and 800 g carrots were provided. For geriatrics, 8–15 kg shoots, 0.8–1.5 kg steamed bread, 500 g apples, and 800 g carrots were provided. The health and ingestion status of each panda was monitored daily by the veterinarians. Fresh fecal samples were collected immediately after defecation, snap-frozen, and shipped to the laboratory on dry ice.

### DNA extraction

According to the manufacturer’s instruction, total genomic DNA was extracted from fecal samples using the PowerFecal DNA Isolation Kit (MOBIO Laboratories, Inc). The concentration and purity of extracted DNA were determined with TBS-380 and NanoDrop 2000, respectively. DNA extract quality was checked on 1% agarose gel.

### Metagenomic sequencing

DNA extract was fragmented to the Covaris M220 ultrasonicator (Covaris, Woburn, USA) for paired-end library construction. A paired-end shotgun library was prepared following the standard Illumina TruSeq™ DNA Sample Prep Kit protocol (Illumina, San Diego, USA). Paired-end sequencing was performed on an Illumina HiSeq4000 platform (Illumina, Inc., San Diego, CA, USA) at Majorbio Bio-Pharm Technology Co., Ltd. (Shanghai, China).

### Quality control of the sequence

Adapter sequences were cut from the 3′ end and the 5′ end of paired-end Illumina reads using SeqPrep (https://github.com/jstjohn/SeqPrep). Low-quality reads with a length < 50 bp or quality value < 20 or having an N base were removed by Sickle (https://github.com/najoshi/sickle). The paired-end reads were compared to the DNA sequence, including GP *Ailuropoda melanoleuca* [79] (i.e., host)*, Daucus carota, Malus domestica, Zea mays, Glycine max*, *Oryza sativa* and Moso Bamboo [80] (i.e., diet) (https://www.ncbi.nlm.nih.gov/genome/) by Burrows-Wheeler Aligner (BWA) [81] (http://bio-bwa.sourceforge.net) and any hit associated with the reads and their mated reads were removed.

### Genome assembly and Texonomical annotation Metagenome dataset

The high quality microbial reads of each fecal sample were processed with IDBA-UD (http://i.cs.hku.hk/~alse/hkubrg/projects/idba_ud/) and bowtie2 (http://bowtie-bio.sourceforge.net/bowtie2/index.shtml) to obtain a part of contigs. In contigs, < 1000 bp were spliced again with Newbler. Open reading frames (ORFs) from each spliced contig were predicted using MetaGene [82] (http://metagene.cb.k.u-tokyo.ac.jp/). For this purpose, a gene with a nucleic acid length of 100 bp or longer was selected and translated into an amino acid sequence using the National Center for Biotechnology Information (NCBI) translation table. The predicted gene sequences from all samples with a 95% sequence identity (90% coverage) were clustered using CD-HIT software [83] (http://www.bioinformatics.org/cd-hit/), and the most extended sequences were selected from each cluster as representative sequences to construct a non-redundant gene catalog. High-quality reads of each sample were compared with the non-redundant “GP gut microbiome gene set” (95% identity) using SOAPalinger [84] (Version 2.22, http://soap.genomics.org.cn/) and the abundance of genes in corresponding samples was calculated. The taxonomic assignment of the non-redundant predicted gene set was aligned to the NCBI NR database with an e-value (cutoff=10^− 5^) using BLASTP [85] (version 2.2.28+, http://blast.ncbi.nlm.nih.gov/Blast.cgi). For each sample, taxonomic sources of genes were annotated by aligning high-quality microbial reads to the non-redundant gene set using Bowtie2 (http://bowtie-bio.sourceforge.net/bowtie2/index.shtml). The abundance of the taxonomic profiles was calculated based on the genus-level classification. We compared the abundance of bacterial genera and results were analyzed by a one-way analysis of variance (ANOVA) using the GLM procedure of SAS (SAS Institute; Cary, NC) and FDR correction by Benjimini and Hochberg method. Differences among means were tested with Duncan’s multiple-range tests. *P* ≤ 0.05 was considered as significant.

### Analysis of antibiotic resistance genes, gene function, and pathway annotations

Using BLASTP [85] (version 2.2.28+, http://blast.ncbi.nlm.nih.gov/Blast.cgi), the non-redundant panda gut microbiome gene set was further searched against the Comprehensive Antibiotic Resistance Database (CARD, https://card.mcmaster.ca) [86] to assess the presence of antibiotic resistance genes. An analysis of the top 30 ARGs to each individual was performed through heatmap in RStudio. Finally, we compared the relative abundance of the top 10 ARGs. Cluster of orthologous groups of proteins (COG) annotation for the representative sequences was performed using BLASTP against the eggNOG database [87, 88] (version 4.5) via BLASTP (BLAST version 2.2.28+) with an e-value (cutoff=1e^− 5^) and the Kyoto Encyclopedia of Genes and Genomes (KEGG) pathway annotation was performed using BLASTP (Version 2.2.28+) against the KEGG database [89] (http://www.genome.jp/keeg/) at an optimized e-value (cutoff of 1e^− 5^). Significant difference tests for ARGs, biosynthesis of antibiotics, amino acid, fatty acid, antimicrobial resistance pathways, and metabolic pathways in the gut microbiome of GPs across the three different age groups were performed by one-way analysis (ANOVA) through the GLM procedure of SAS (SAS Institute; Cary, NC) 9.4 version followed by FDR correction test of Benjimini and Hochberg. Differences among means were tested with Duncan’s multiple range tests. *P* ≤ 0.05 is considered significant. Figures were visualized using GraphPad prism.

### Network analysis

A network was created using the relative abundance data of bacterial genera and ARGs from 60 samples. The analysis was performed in the SPSS V21 using the stats package. A correlation coefficient with numeric values, either ≥ 0.8 or ≤ − 0.8, and the significance of *P* ≤ 0.05 was considered statistically robust. Only significant pairwise relationships were used while constructing networks, with each node representing bacterial genera and ARGs and each edge representing a significant pairwise association between them. A positive correlation between bacterial genera and ARGs denoted similar abundance patterns. In contrast, a negative relationship was characterized as opposite abundance patterns. Interacting nodes within networks represented co-occurrence across samples. Similarly, two more networks were created using the relative abundance data of pathways in biosynthesis of antibiotics, metabolic pathways, and the top ten ARGs. Networks were visualized using Cytoscape 3.6.0.

### Antimicrobial susceptibility testing

We isolated 11 *Streptococcus* strains from collected fecal samples and tested against 13 antimicrobials (Additional file 3: Table S2 a,b). The susceptibilities of all the isolates were determined independently against beta-lactam (Penicillin 10 μg, Ampicillin 10 μg, Ceftriaxone 5 μg); Macrolides (Erythromycin 15 μg, Azithromycin 15 μg, Clarithromycin 5 μg); Lincosamide (Clindamycin 2 μg); Quinolones (Levofloxocin 5 μg, Ofloxacin 5 μg); Ansamycin (Rifampin 5 μg); Glycopeptides (Vancomycin 30 μg); Miscellaneous (Chloramphenicol 30 μg); Carbapenems (Meropenem 10 μg) using the standard Kirby–Bauer disk diffusion method [90]. Colony suspension, equivalent to a 0.5 McFarland standard, were prepared using colonies from an overnight (18-20 h) sheep blood agar plate at 35±2 °C.Antimicrobial disks (Oxoid Ltd., Basingstoke, UK) were placed on the MHA plates (Becton Dickinson, Franklin Lakes, NJ) with 5% sheep blood and incubated aerobically at 35±2 °C for 20–24 h. The inhibition zones’ diameter surrounding the antimicrobial disks was interpreted according to Clinical and Laboratory Standards Institute guidelines (CLSI, M100, 30th Edition, 2020). Quality control for susceptibility testing was done using *S. pneumoniae* ATCC® 49619.

## Supplementary Information

**Additional file 1: Figure S1.** Details of collected samples of three different age groups of GPs. **Figure S2a.** The abundance of different antibiotic families in fecal samples of 60 GPs. **Figure S3.** The abundance (%) of top 30 ARGs in each sample of three different age groups of GPs. **Figure S4.** An average abundance of approximately 20 genera in fecal samples of 60 GPs. **Figure S5.** Abundance of the top three bacteria at the genus level in three different groups of GPs juvenile, adult, and geriatric. **Figure S6.** The network analysis of 84 genera with an average abundance of ≥ 0.01% based on the Pearson coefficient of correlation. **Figure S7.** Quantitative analysis of the proteome of the gut microbiota of captive GPs. **Figure S8.** The increasing and decreasing level of *Escherichia* and *Streptococcus* in three different groups (juvenile, adult, and geriatric) of GPs and the significant difference of abundance of these genera among these groups. **Figure S9.** (A) Biosynthesis of Antibiotic and (B) Resistant Pathway.

**Additional file 2: Table S1.** The 10 most abundant ARGs type presented in each Group (juvenile, adult and geriatric) of GPs. **Table S3.** Correlation analysis of Bacterial genus and ARGs. **Table S4.** Correlation analysis of Metabolic pathway, pathways in biosynthesis of antibiotic and ARGs.

**Additional file 3: Table S2.** a and b Details about the Antimicrobial susceptibility in *Streptococcus.*

**Additional file 4: Figure S2b.** The abundance of 570 ARGs ranged from 0.0001 to 3.6%. Highlighted area indicating top most abundant ARGs.

## Data Availability

The datasets used and/or analyzed during the current study available from the corresponding author on reasonable request.

## Abbreviations

ANOVA: Analysis of variance
ARGs: Antibiotic resistance gene
CCRCGP: China Conservation and Research Center for the Giant Panda
CARD: Comprehensive Antibiotic Resistance Database
CLSI: Clinical and Laboratory Standards Institute guidelines
COG: Cluster of orthologous groups
GP: Giant panda
H_2_O_2_: Hydrogen peroxide
KEGG: Kyoto Encyclopedia of Genes and Genomes
