# Supplementary material for: Metagenomic analysis revealed a wide distribution of antibiotic resistance genes and biosynthesis of antibiotics in the gut of giant pandas
Source: BMC Microbiol. 2021 Jan 7;21:15. doi: 10.1186/s12866-020-02078-x (PMC7792088; doi:10.1186/s12866-020-02078-x)

**Addition File 1**

**
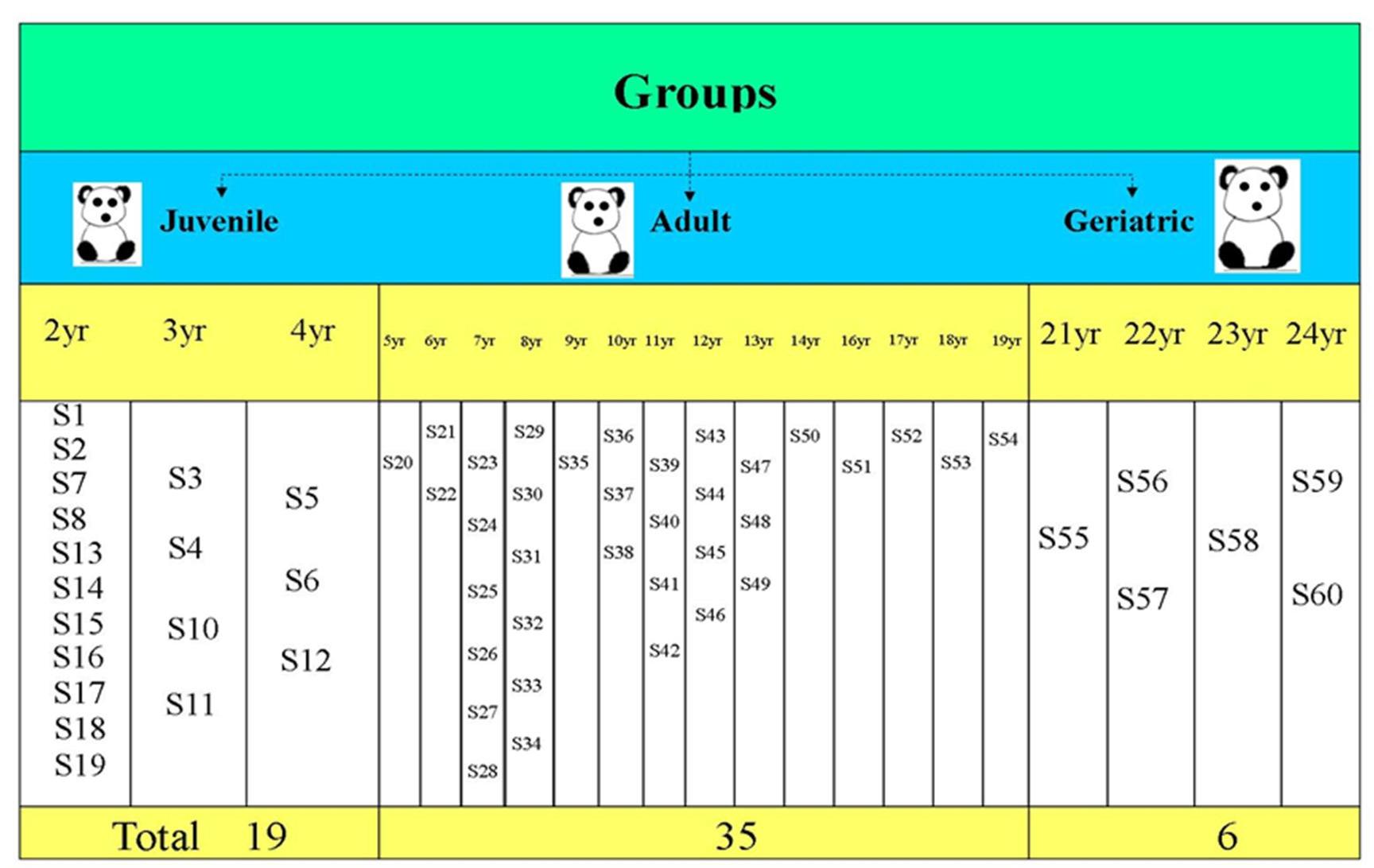
**

**Figure S1**: Details of collected samples of three different age groups of GPs.


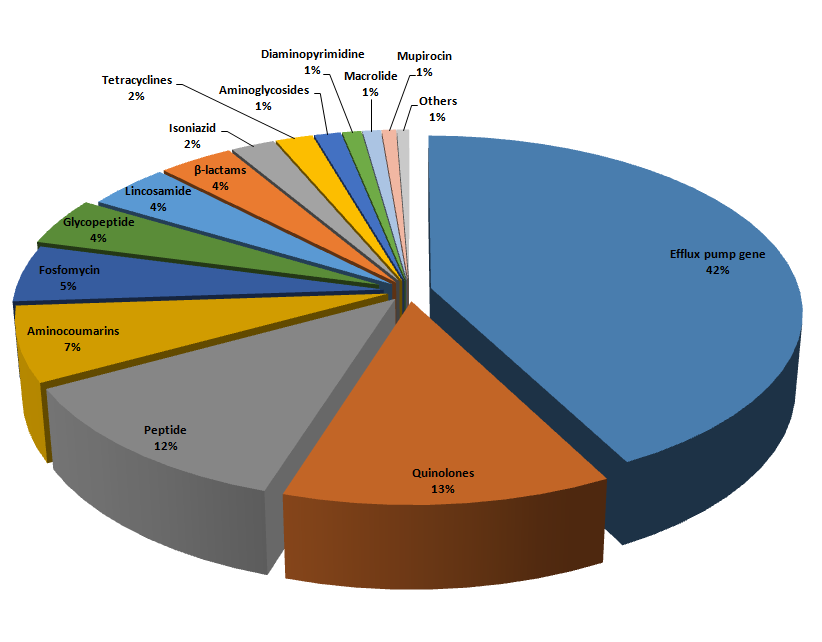


**Figure S2a**: The abundance of different antibiotic families in fecal samples of 60 GPs


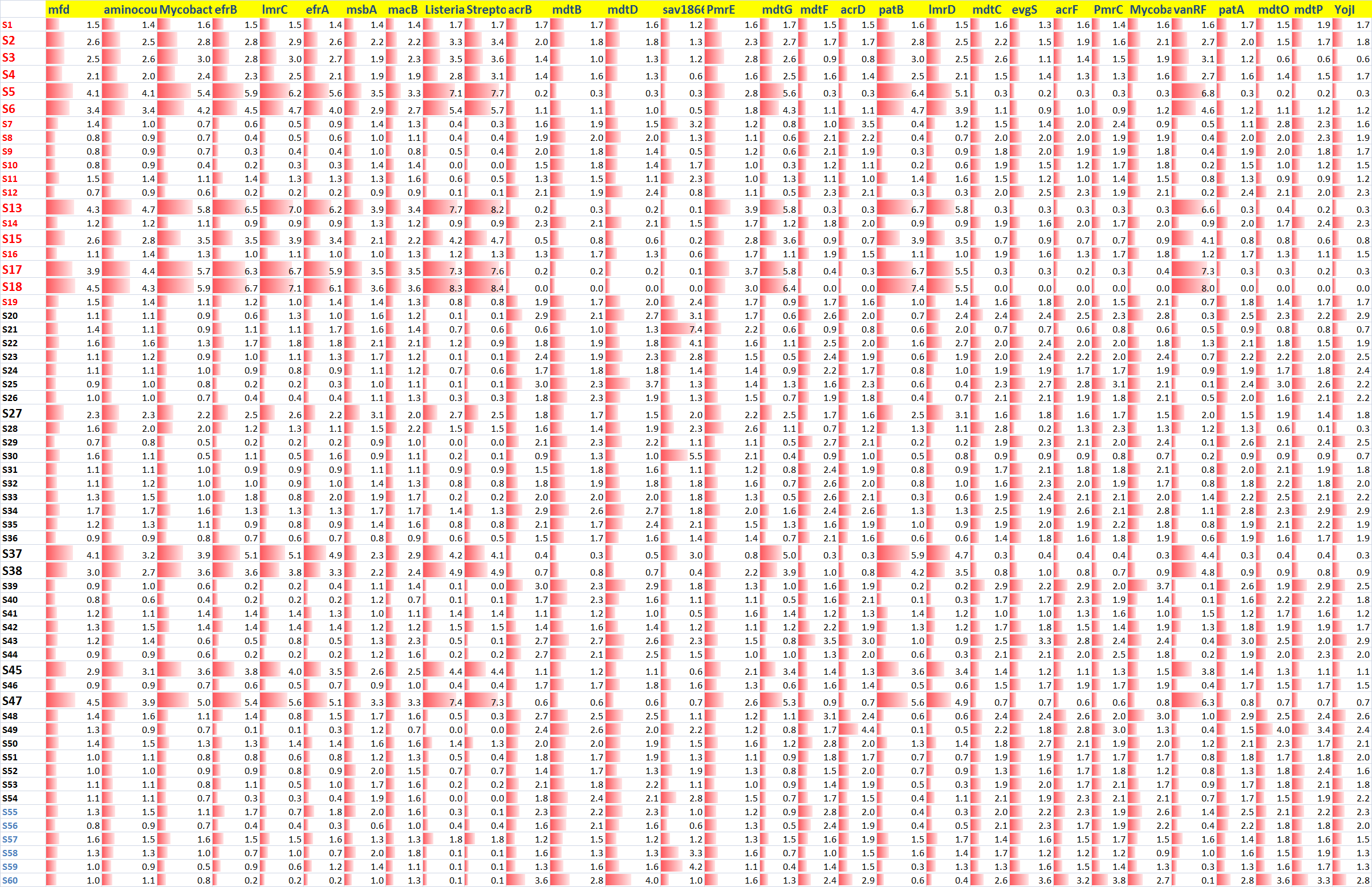


**Figure S3**: The abundance (%) of top 30 ARGs in each sample of three different age groups of GPs. From S1 to S19 are juvenile, from S20 to S54 are adult and from S55 to S60 are geriatric GPs.


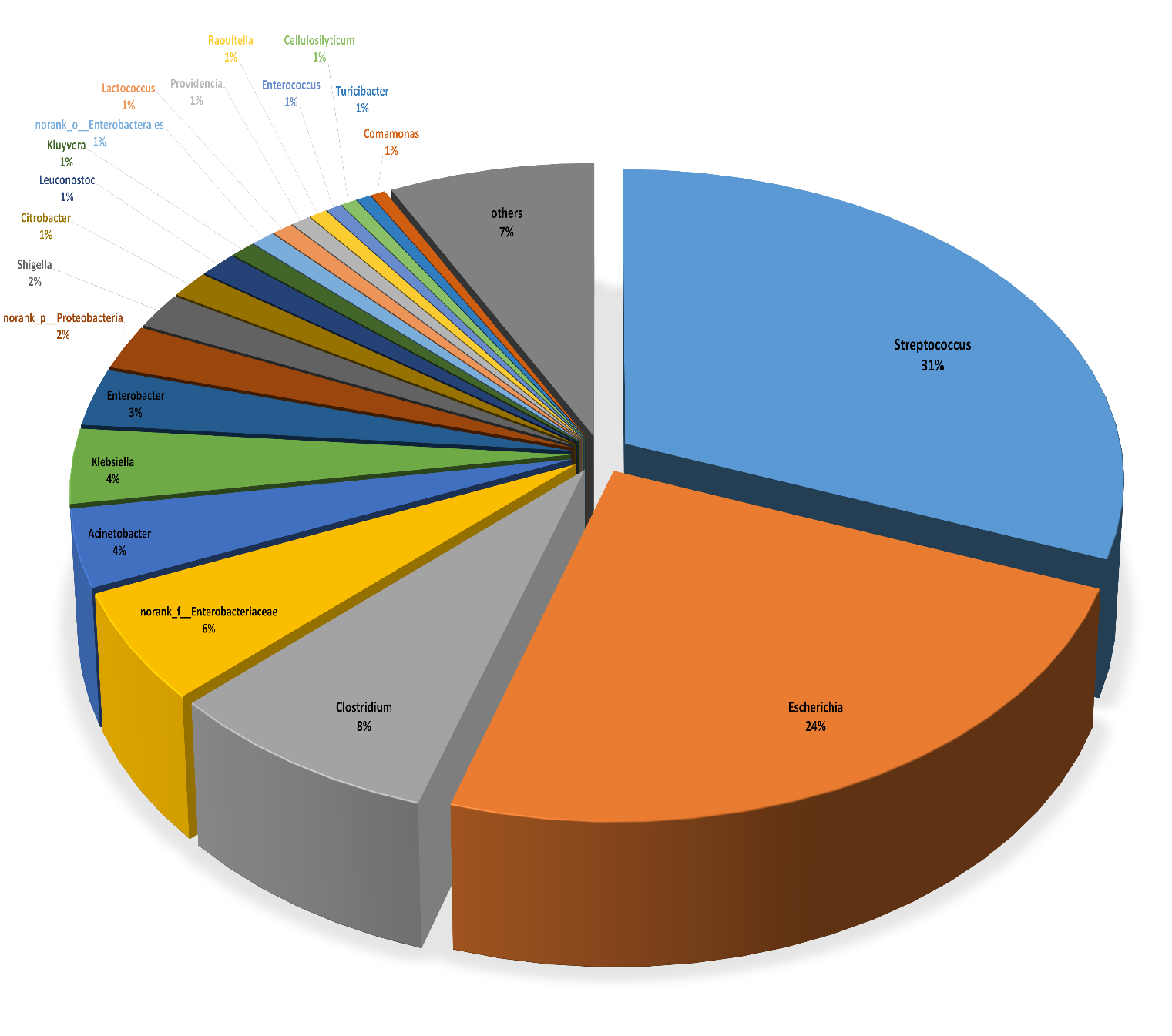


**Figure S4**: An average abundance of approximately 20 genera in fecal samples of 60 GPs.


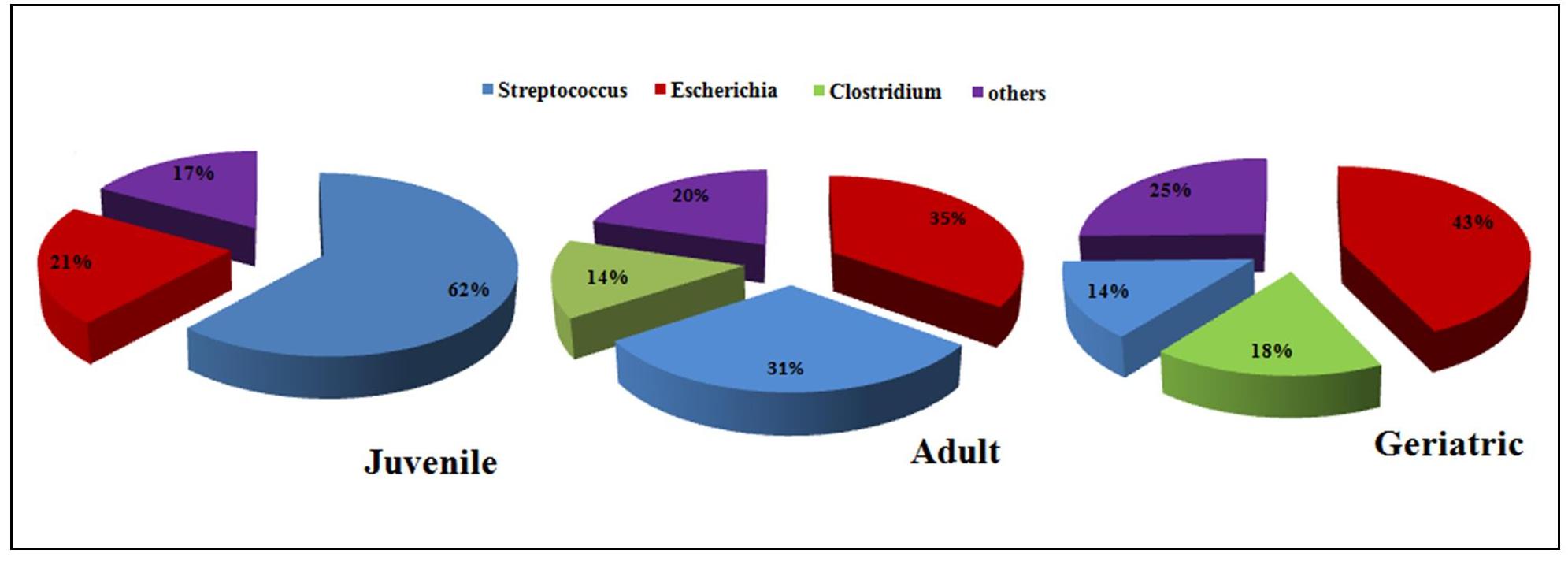


**Figure S5**: Abundance of the top three bacteria at the genus level in three different groups of GPs juvenile, adult, and geriatric.


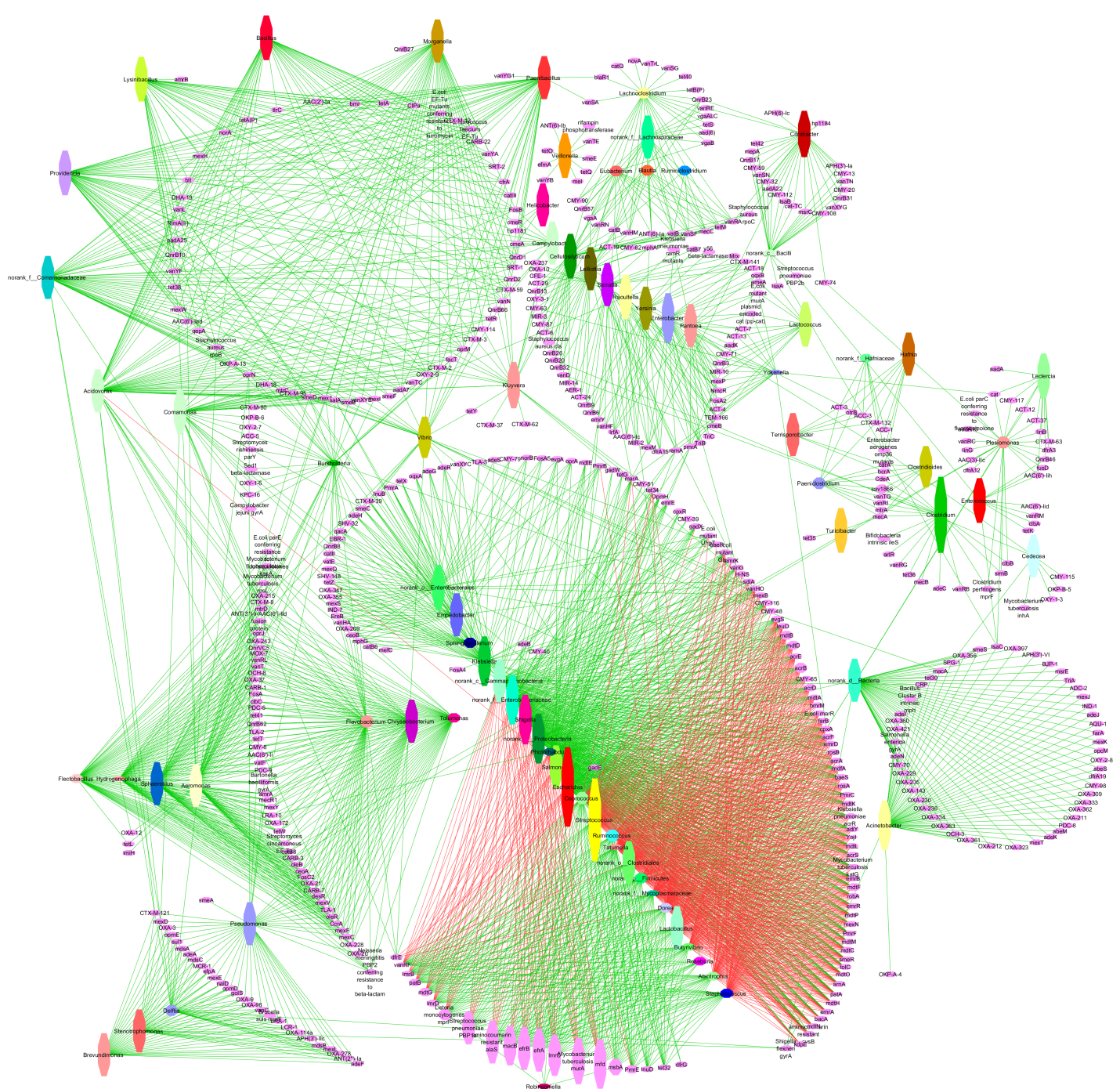


**Figure S6:** The network analysis of 84 genera with an average abundance of ≥ 0.01% based on the Pearson coefficient of correlation. The network analysis revealing the co-occurrence patterns between 84 genera and ARGs. Nodes of different colors represent different genera whereas nodes of pink color represent ARGs. A connection represents a strong (Spearman’s correlation coefficient R^2^ > 0.8- 0.9) and significant (*p-value* < 0.05) correlation. Edges colored according to the correlation coefficient (green and red color represent positive and negative correlation) and node size weighted according to the relative abundance of ARGs and genera.


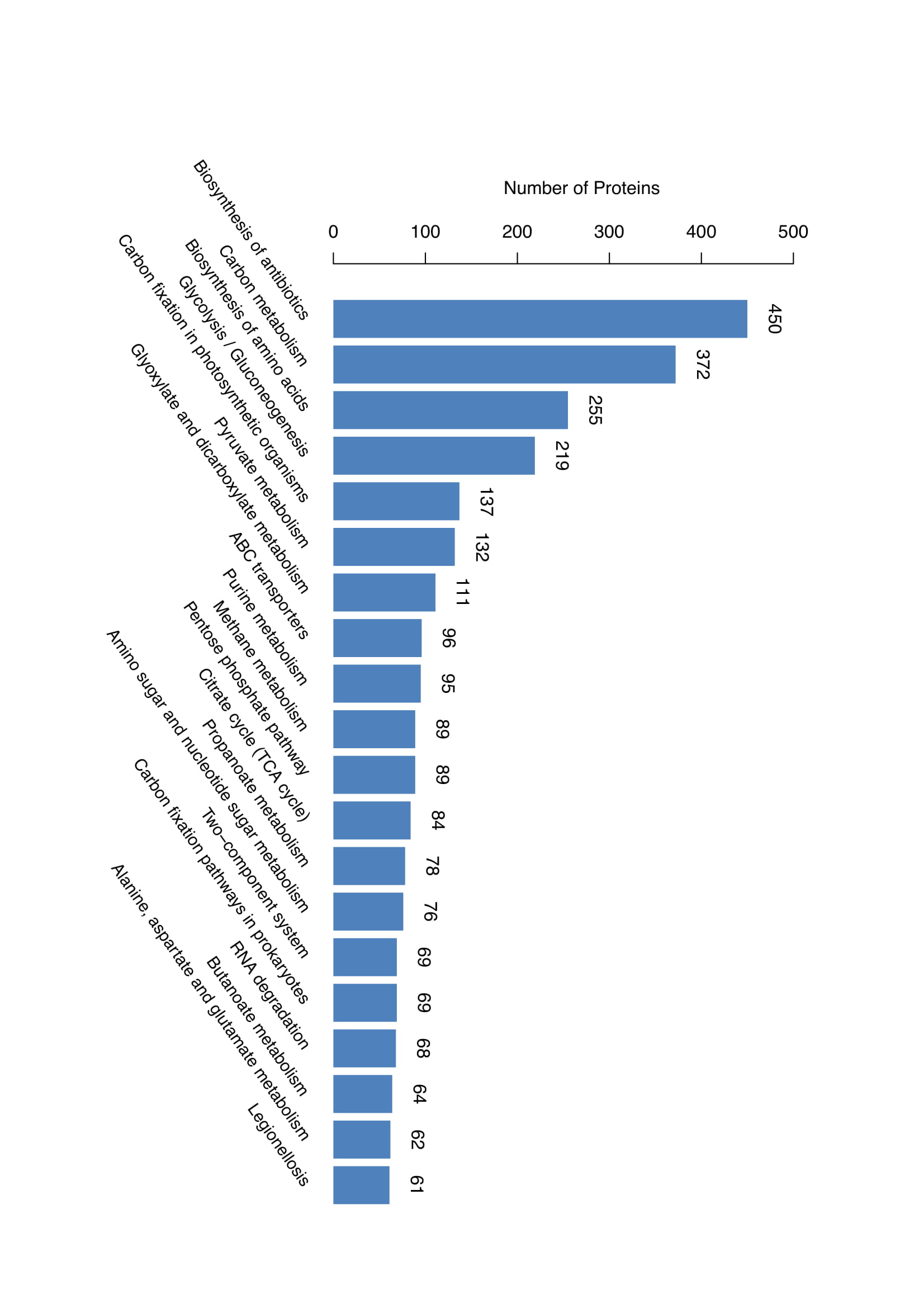


**Figure S7:** Quantitative analysis of the proteome of the gut microbiota of captive GPs.


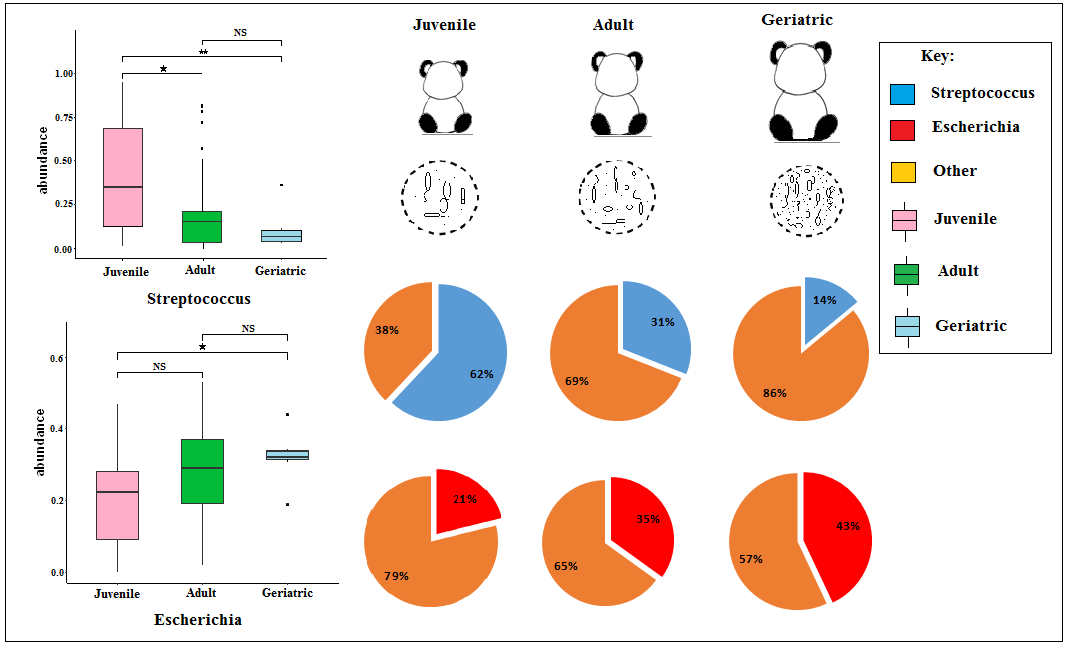


**Figure S8:** The increasing and decreasing level of *Escherichia* and *Streptococcus* in three different groups (juvenile, adult, and geriatric) of GPs and the significant difference of abundance of these genera among these groups.

**Figure S9: (A) Biosynthesis of Antibiotic and (B) Resistant Pathway**

1. **Biosynthesis of Antibiotic**


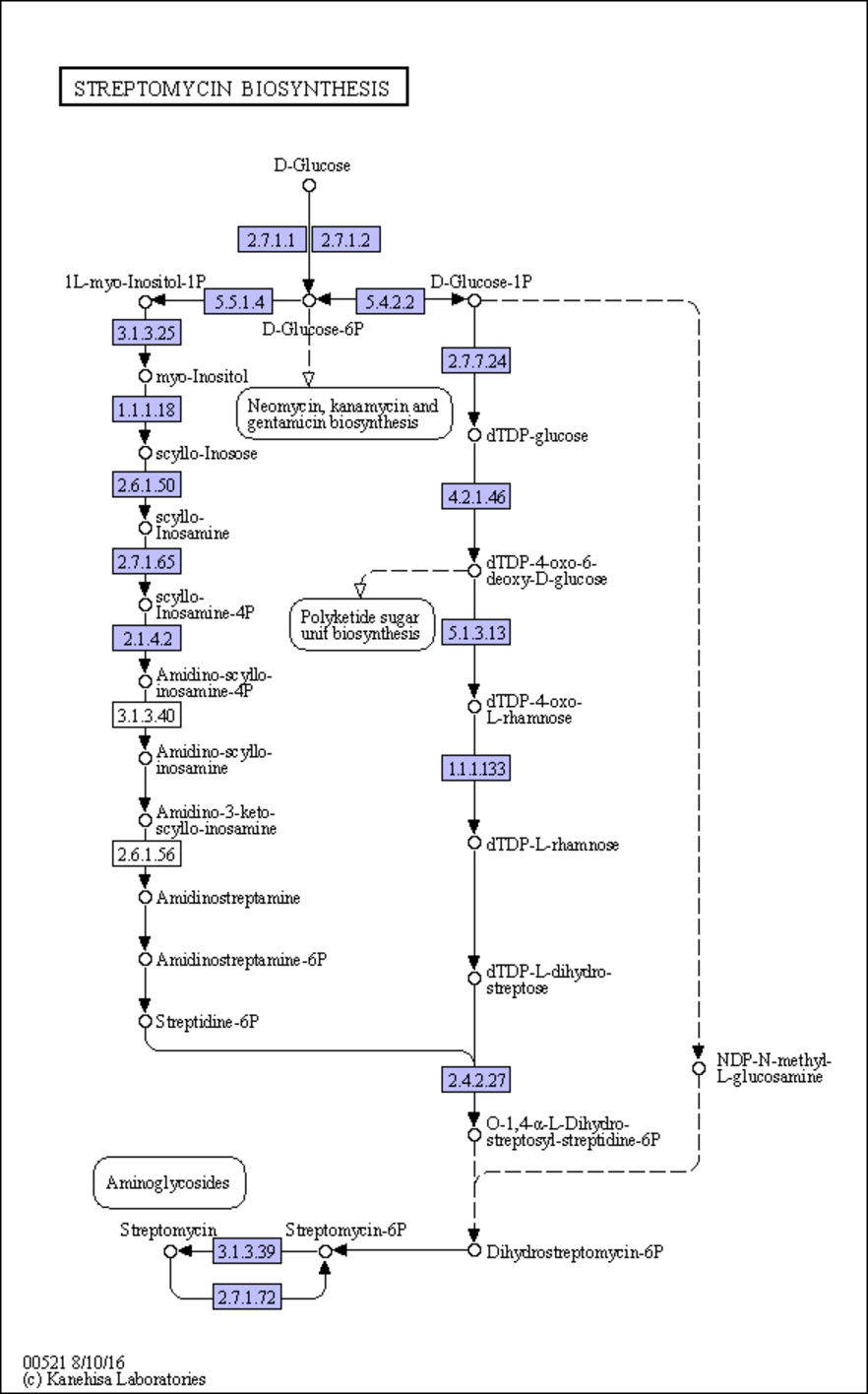


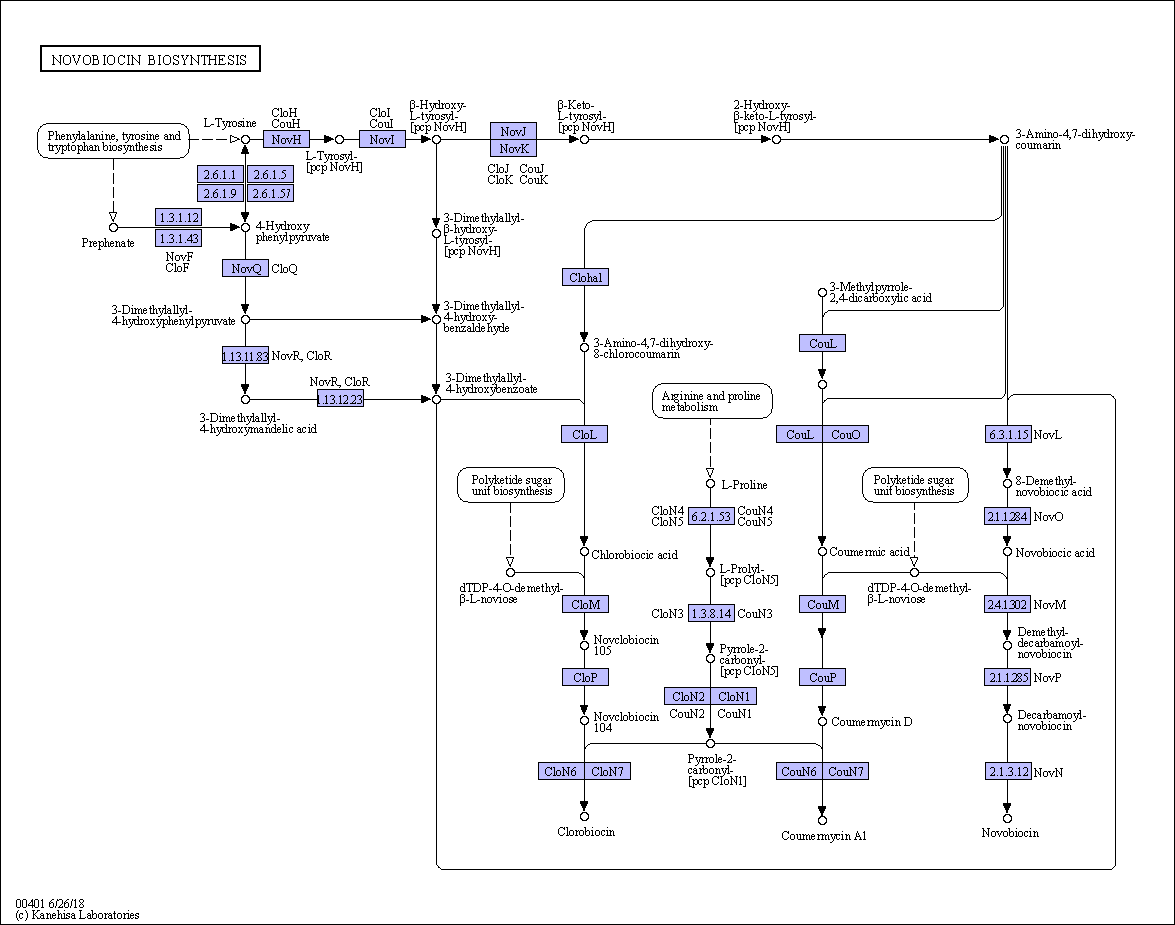


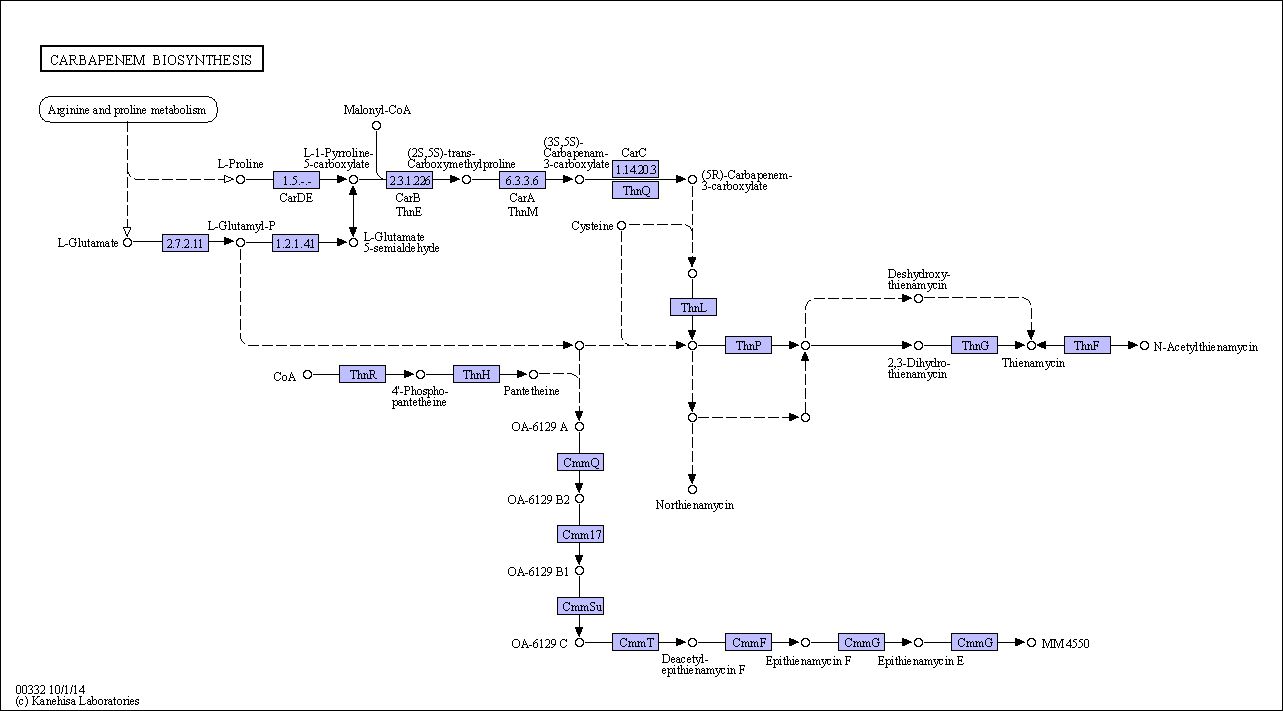


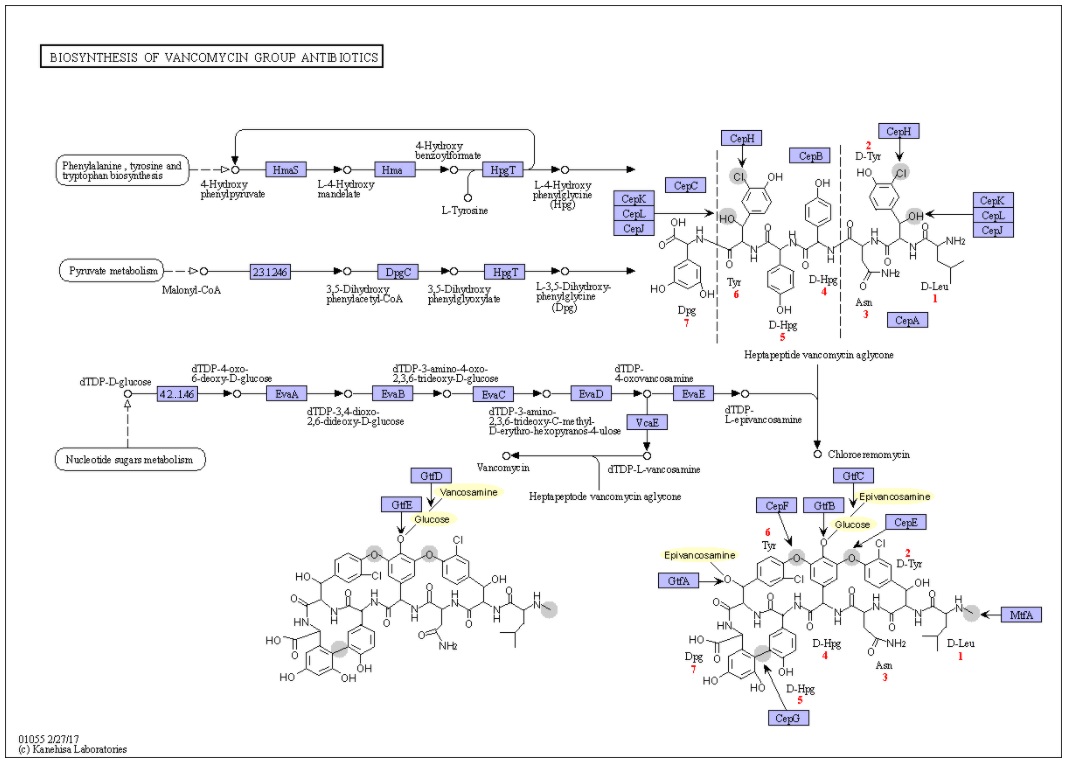


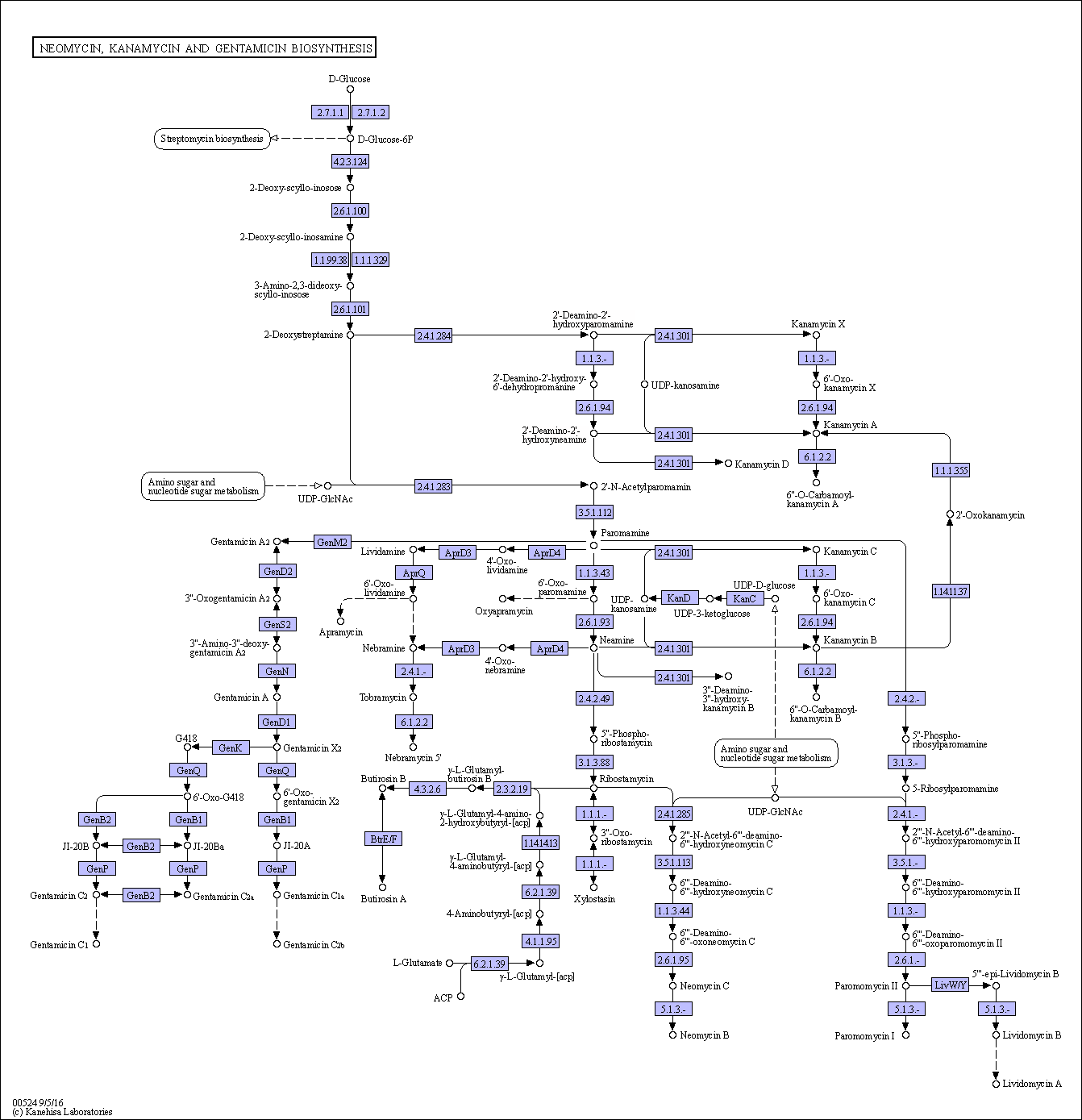


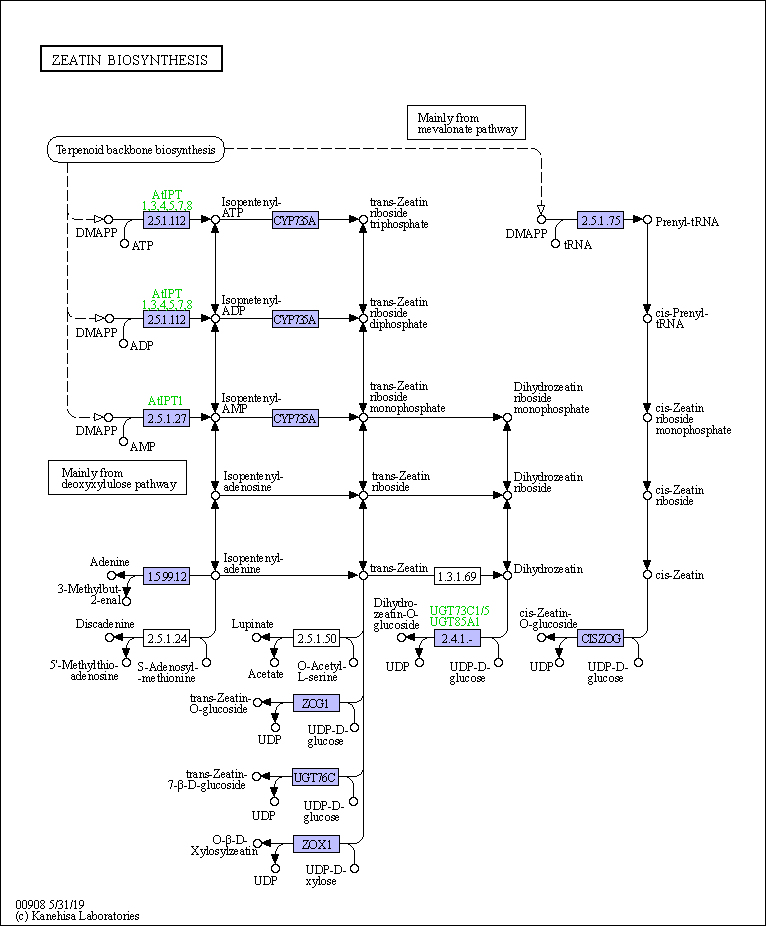


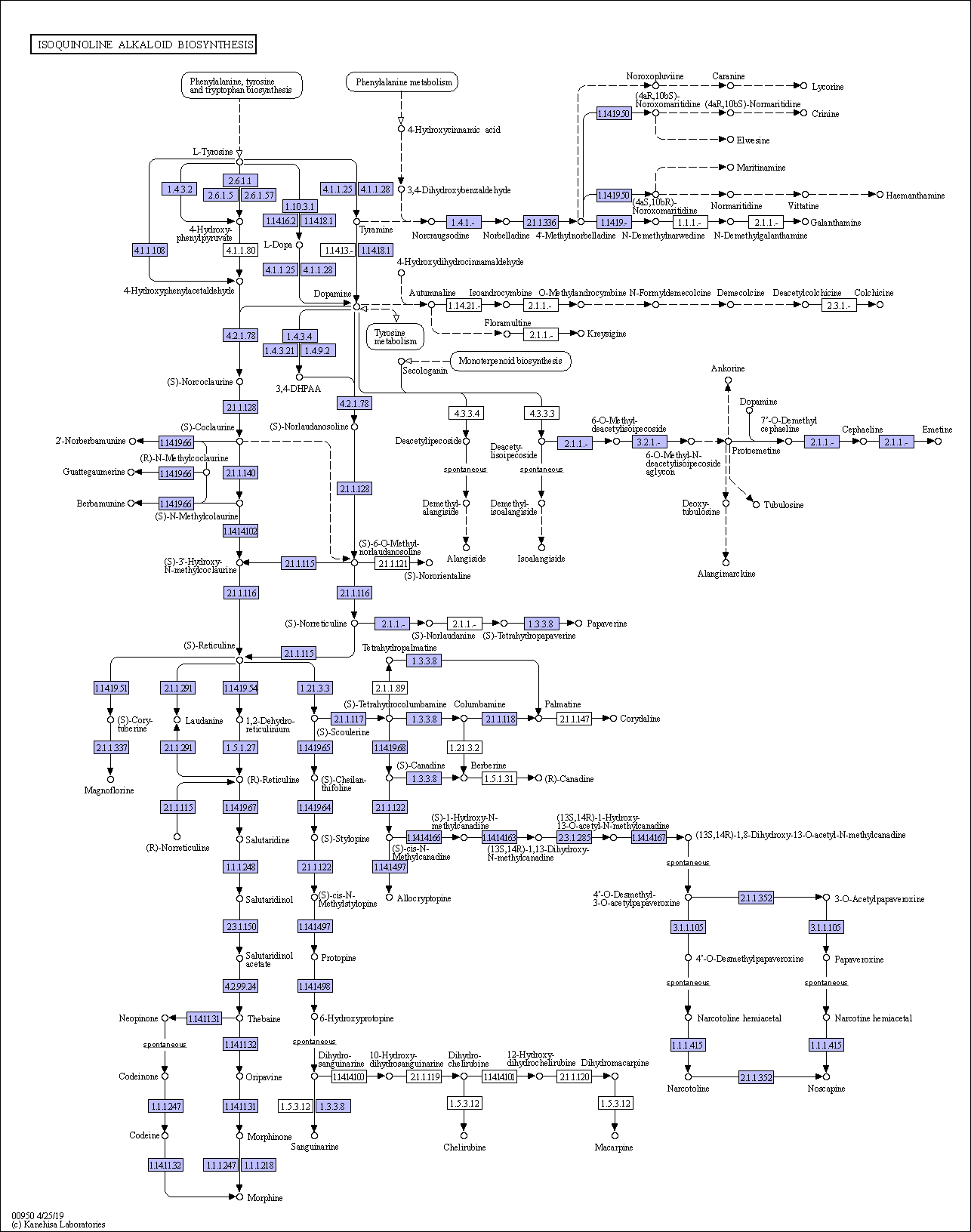


1. **Resistant Pathway**


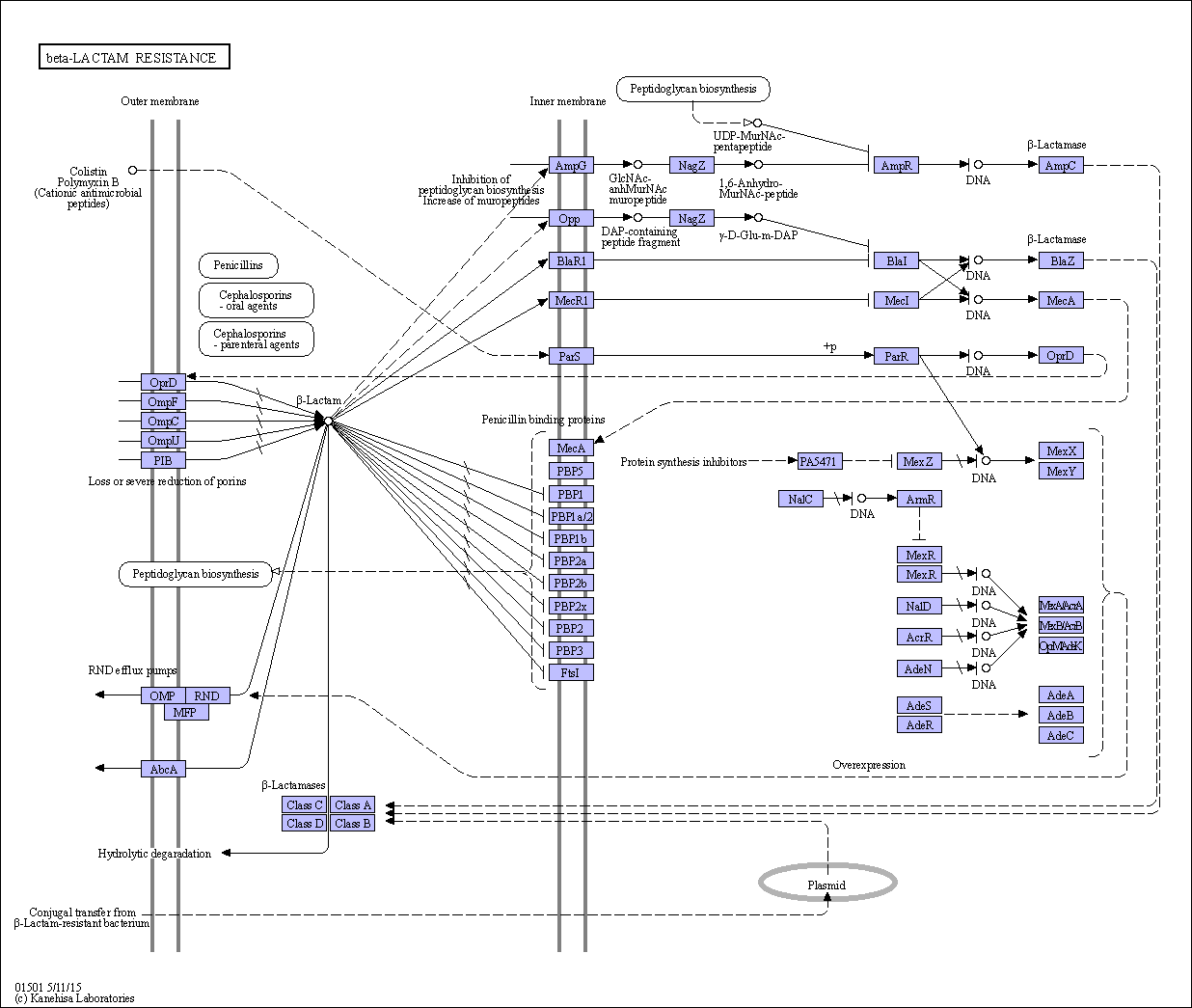


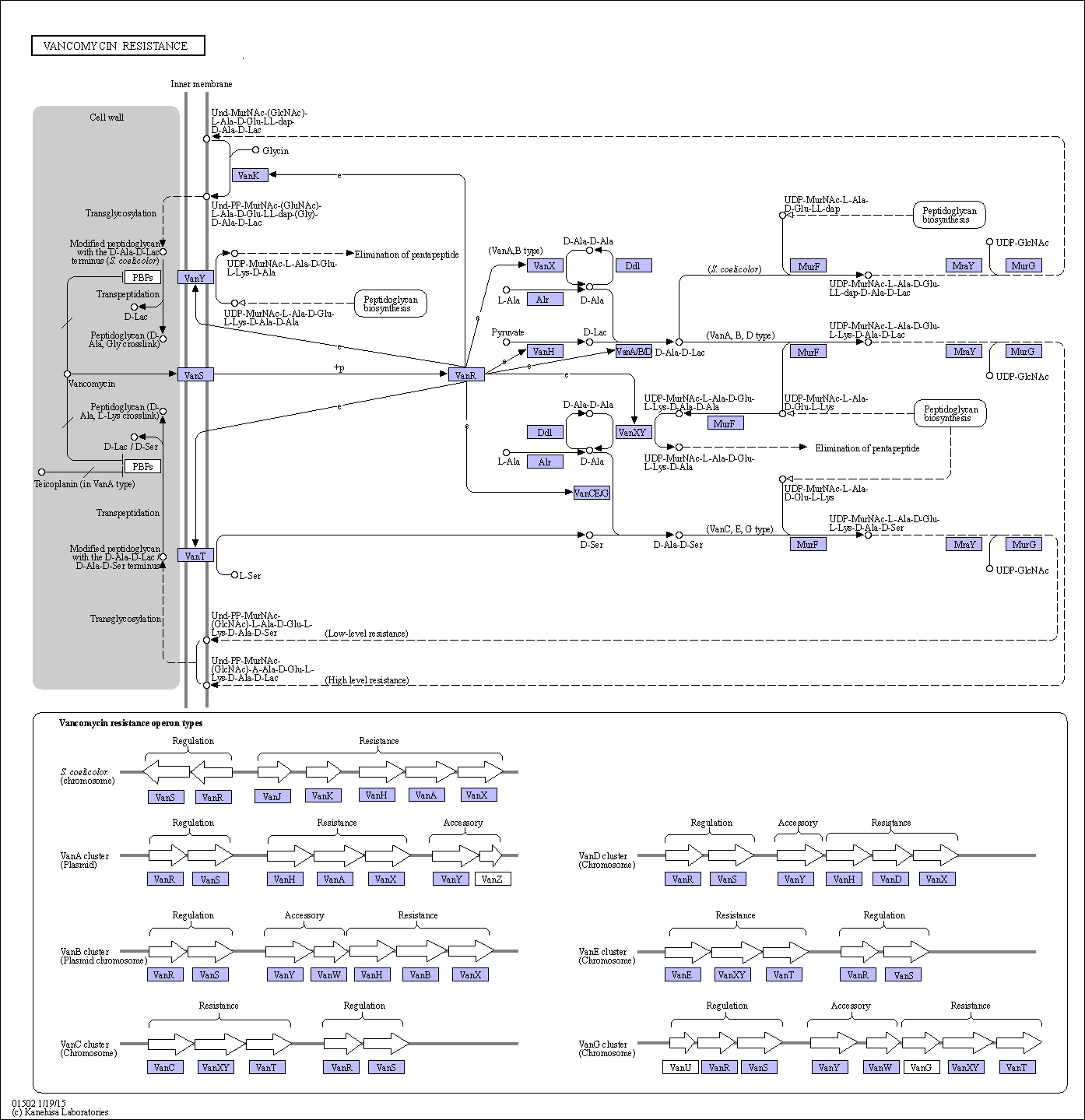


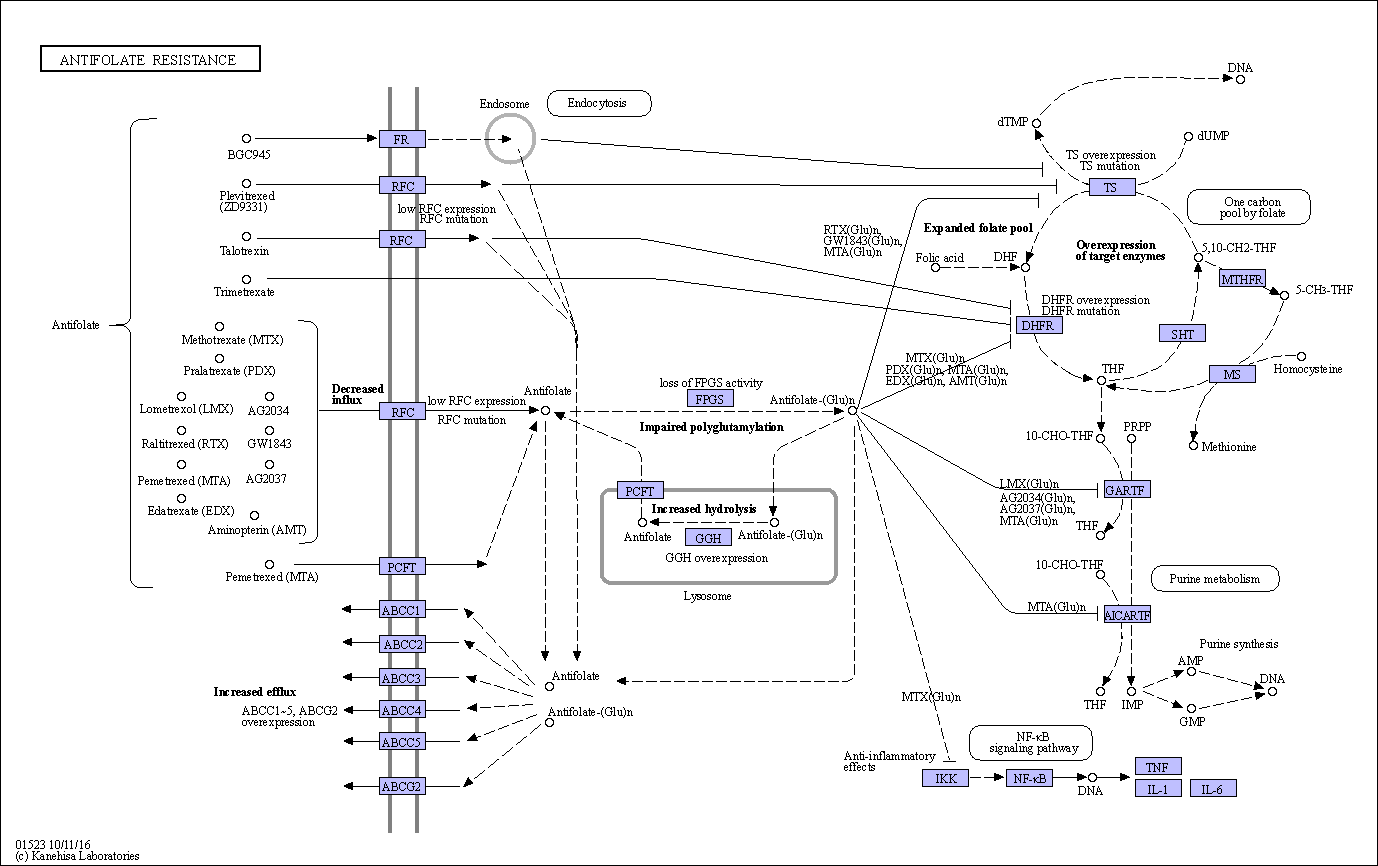

Supplement: Supplementary file 1 — Additional file 1: Figure S1. Details of collected samples of three different age groups of GPs. Figure S2a. The abundance of different antibiotic families in fecal samples of 60 GPs. Figure S3. The abundance (%) of top 30 ARGs in each sample of three different age groups of GPs. Figure S4. An average abundance of approximately 20 genera in fecal samples of 60 GPs. Figure S5. Abundance of the top three bacteria at the genus level in three different groups of GPs juvenile, adult, and geriatric. Figure S6. The network analysis of 84 genera with an average abundance of ≥ 0.01% based on the Pearson coefficient of correlation. Figure S7. Quantitative analysis of the proteome of the gut microbiota of captive GPs. Figure S8. The increasing and decreasing level of Escherichia and Streptococcus in three different groups (juvenile, adult, and geriatric) of GPs and the significant difference of abundance of these genera among these groups. Figure S9. (A) Biosynthesis of Antibiotic and (B) Resistant Pathway. [file 12866_2020_2078_MOESM1_ESM.docx]
