# Supplementary figures and images for: Metagenomic analysis revealed a wide distribution of antibiotic resistance genes and biosynthesis of antibiotics in the gut of giant pandas
Source: BMC Microbiol. 2021 Jan 7;21:15. doi: 10.1186/s12866-020-02078-x (PMC7792088; doi:10.1186/s12866-020-02078-x)

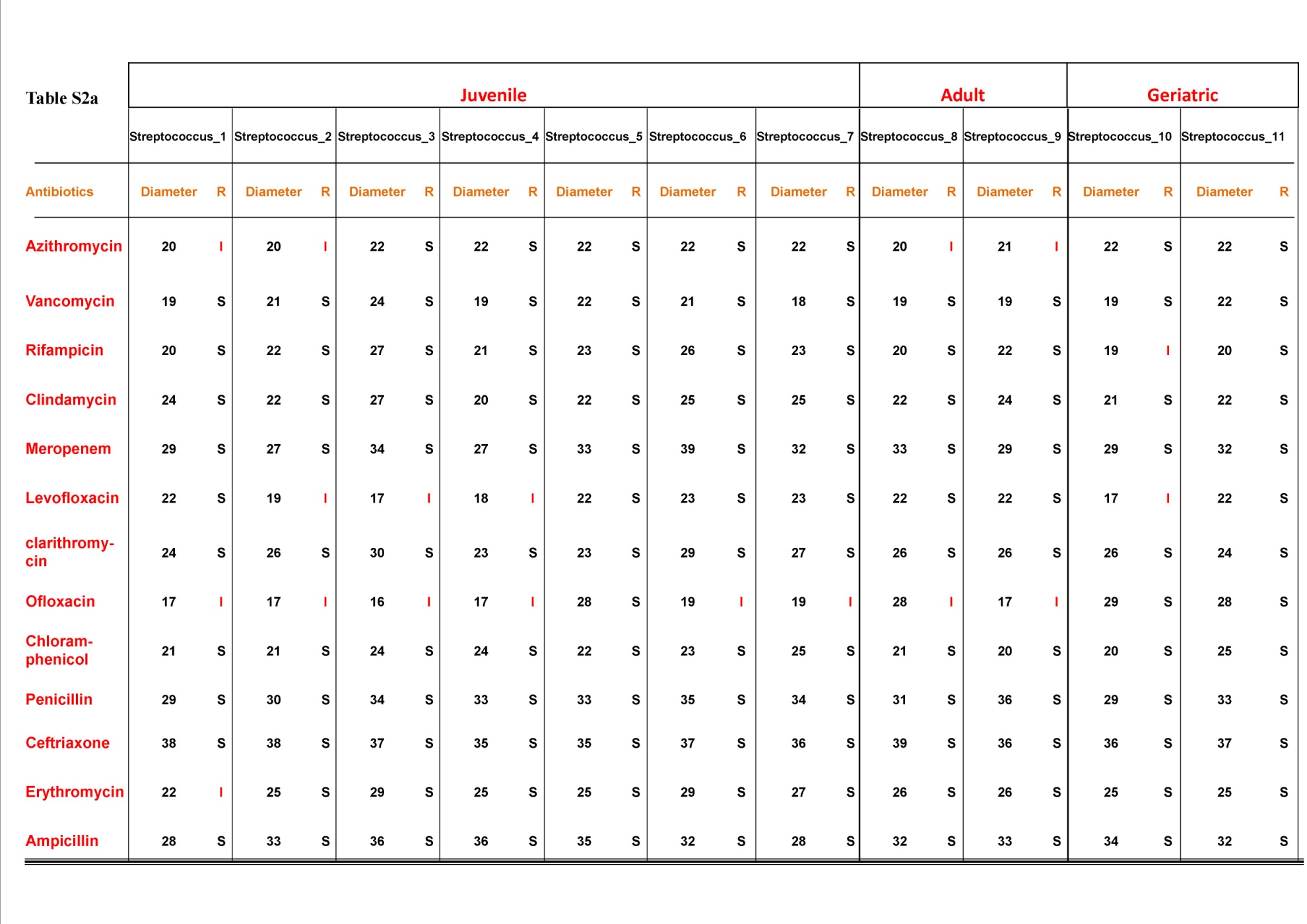


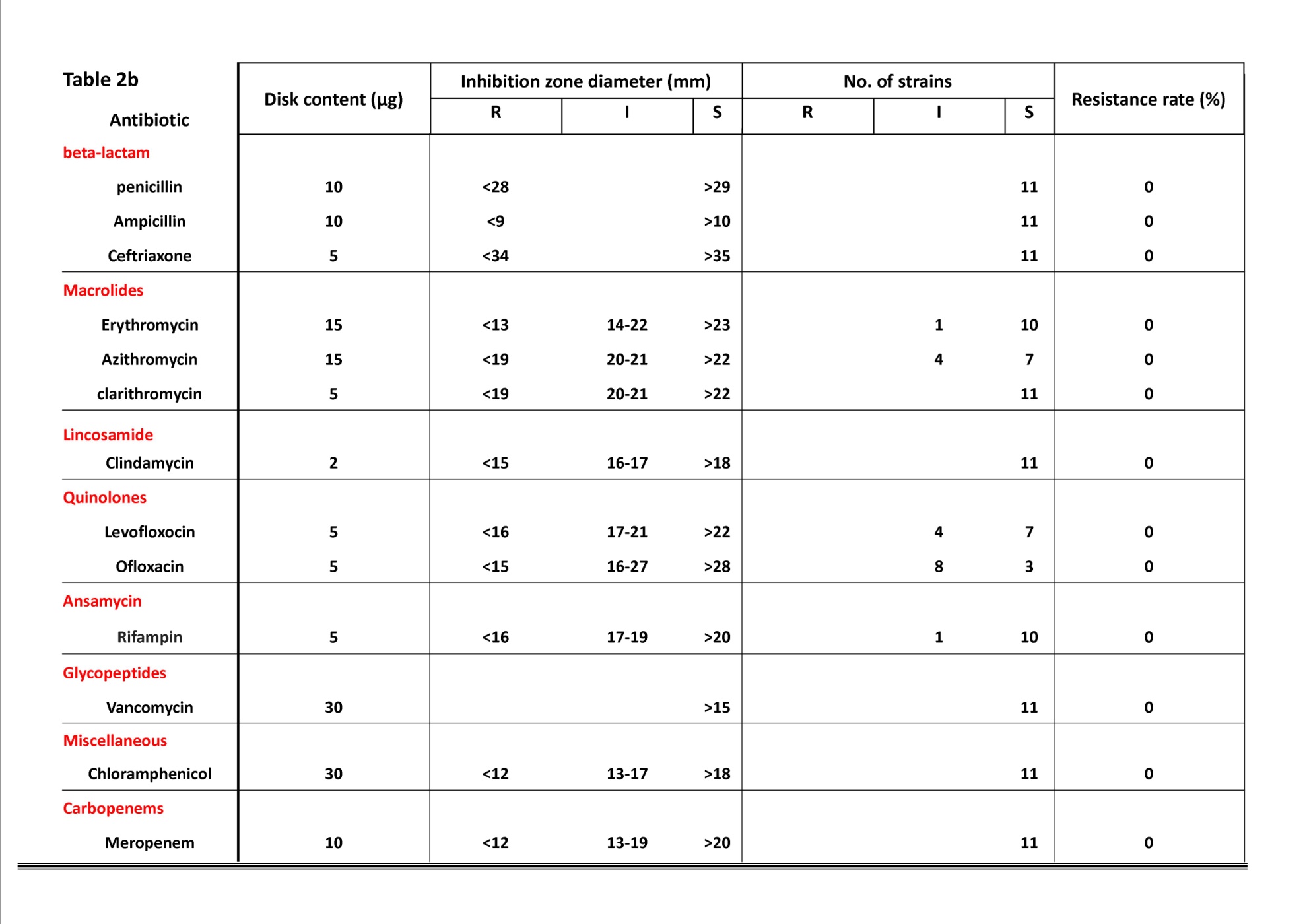

Supplement: Supplementary file 3 — Additional file 3: Table S2. a and b Details about the Antimicrobial susceptibility in Streptococcus. [file 12866_2020_2078_MOESM3_ESM.docx]
