# Supplementary material for: Metagenomic analysis revealed a wide distribution of antibiotic resistance genes and biosynthesis of antibiotics in the gut of giant pandas
Source: BMC Microbiol. 2021 Jan 7;21:15. doi: 10.1186/s12866-020-02078-x (PMC7792088; doi:10.1186/s12866-020-02078-x)
Supplement: Supplementary file 4 — Additional file 4: Figure S2b. The abundance of 570 ARGs ranged from 0.0001 to 3.6%. Highlighted area indicating top most abundant ARGs. [file 12866_2020_2078_MOESM4_ESM.pdf]

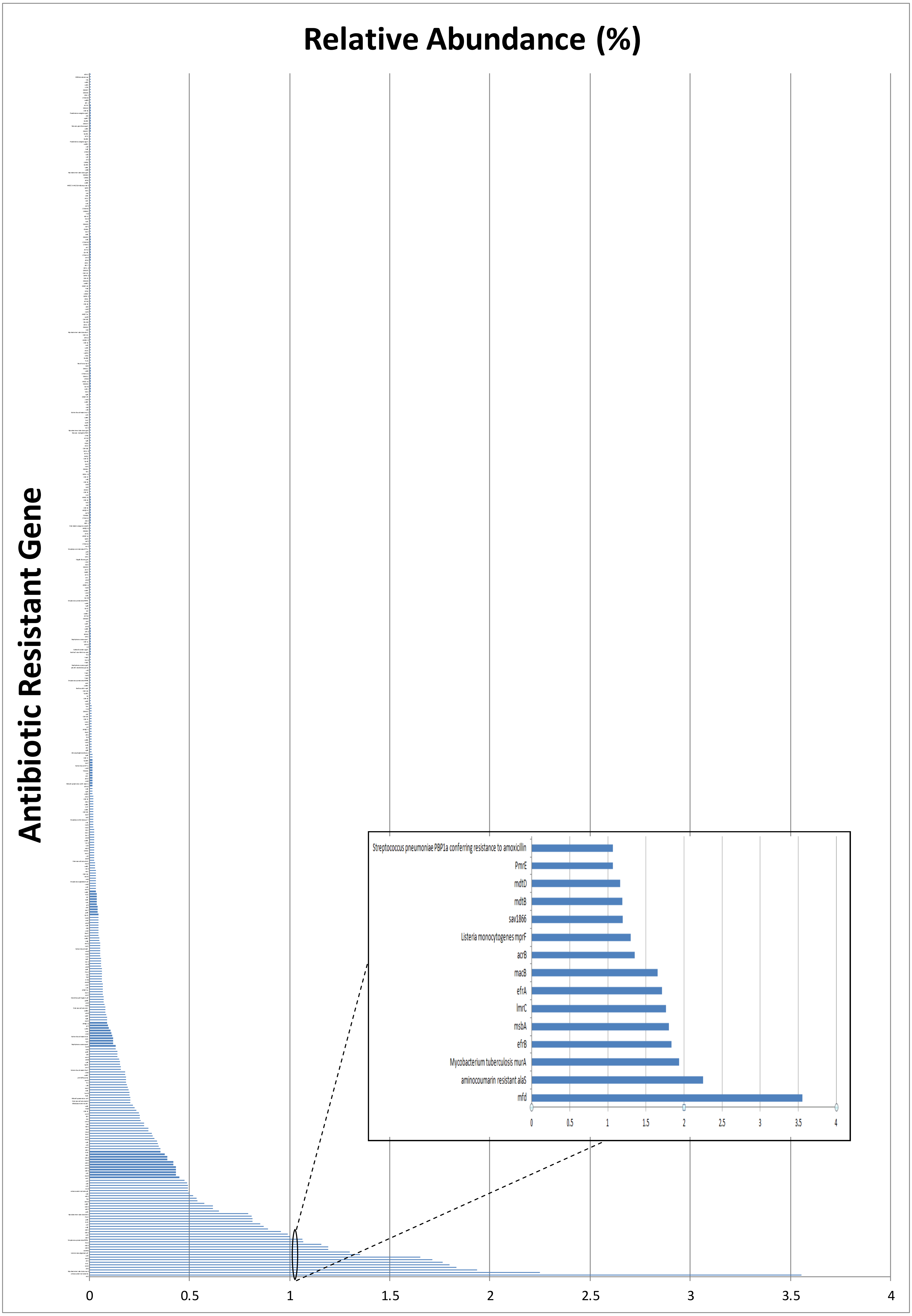

Figure S2b: The abundance of 570 ARGs ranged from 0.0001 % to 3.6%. Highlighted area indicating top most abundant ARGs.
